# Whole-exome sequencing of individuals from an isolated population under extreme conditions implicates rare risk variants of schizophrenia

**DOI:** 10.1038/s41398-024-02984-y

**Published:** 2024-06-29

**Authors:** Lei Chen, Yang Du, Yang Hu, Xue-Song Li, Yuewen Chen, Yong Cheng

**Affiliations:** 1 Key Laboratory of Ethnomedicine of Ministry of Education, Center on Translational Neuroscience, School of Pharmacy, Minzu University of China, Beijing, China; 2 College of Life and Environmental Sciences, Minzu University of China, Beijing, China; 3 The Third People’s Hospital of Foshan, Foshan, China; 4 Chinese Academy of Sciences Key Laboratory of Brain Connectome and Manipulation, Shenzhen Key Laboratory of Translational Research for Brain Diseases, The Brain Cognition and Brain Disease Institute, Shenzhen Institute of Advanced Technology, Chinese Academy of Sciences; Shenzhen–Hong Kong Institute of Brain Science–Shenzhen Fundamental Research Institutions, Shenzhen, Guangdong 518055 China; 5 Guangdong Provincial Key Laboratory of Brain Science, Disease and Drug Development, HKUST Shenzhen Research Institute, Shenzhen, Guangdong 518057 China; 6 Institute of National Security, Minzu University of China, Beijing, China

**Keywords:** Schizophrenia, Genomics

## Abstract

Schizophrenia (SCZ), which affects approximately 1% of the world’s population, is a global public health concern. It is generally considered that the interplay between genes and the environment is important in the onset and/or development of SCZ. Although several whole-exome sequencing studies have revealed rare risk variants of SCZ, no rare coding variants have been strongly replicated. Assessing isolated populations under extreme conditions might lead to the discovery of variants with a recent origin, which are more likely to have a higher frequency than chance to reflect gene-environment interactions. Following this approach, we examined a unique cohort of Tibetans living at an average altitude above 4500 meters. Whole-exome sequencing of 47 SCZ cases and 53 controls revealed 275 potential novel risk variants and two known variants (*12:46244485: A/G and 22:18905934: A/G*) associated with SCZ that were found in existing databases. Only one gene (*C5orf42*) in the gene-based statistics surpassed the exome-wide significance in the cohort. Metascape enrichment analysis suggested that novel risk genes were strongly enriched in pathways relevant to hypoxia, neurodevelopment, and neurotransmission. Additionally, 47 new risk genes were followed up in Han sample of 279 patients with SCZ and 95 controls, only *BAI2* variant appearing in one case. Our findings suggest that SCZ patients living at high altitudes may have a unique risk gene signature, which may provide additional information on the underlying biology of SCZ, which can be exploited to identify individuals at greater risk of exposure to hypoxia.

## Introduction

Schizophrenia (SCZ), which affects approximately 1% of people worldwide [1, 2], is a chronic and severe psychotic disorder thought to have a strong genetic component in which patients typically display auditory hallucinations, delusions, emotional passivation, social withdrawal, and cognitive impairment [3]. Family and twin studies have consistently reported a high heritability of 70–80% for SCZ [4, 5], but only a few points of genetic variance in SCZ have been previously explained at the molecular level. Several common loci deeply influence susceptibility to genetic etiology, for instance, DISC1 [6] and NRG1 [7]. However, specific loci are unable to account for most of the heritability of SCZ. Although roughly 33–50% of the genetic risk of SCZ can be captured by current genome-wide association studies [8], a substantial part of the estimated heritability is still unknown. Thus, an approach that accounts for the high heritability of SCZ is important for investigating genetic susceptibility to SCZ.

To that end, whole-exome sequencing (WES) studies have proven successful in disentangling complex phenotypes by identifying causative genetic mutations [9]. In particular, WES studies have identified rare variants and mutants that significantly influence the risk genes for autism [10]. Although family-based WES has been successfully used to study risk genes for SCZ, only a few studies on this topic have been reported. For example, using WES, Daniel et al. found that de novo mutations in protein coding genes explain only a small fraction of SCZ risk [11]. Another study using WES reported disruptive de novo variants screened from 591 exome-sequenced SCZ cases and their parents [12]. Although family based studies can exclude some confounding factors such as population structure differences, these studies cannot explain the relationship between multiple factors and SCZ.

It is well established that gene-environment interactions are important for the development of SCZ; however, the mechanism(s) by which environmental factors influence SCZ-related genes remains poorly understood [13]. Importantly, environmental risk factors can function on an individual level or on a population level, and their effects can either be the direct or indirect cause of the risk increase. Generally, there are many limitations to studying environmental risk factors for SCZ which are very difficult to measure. For instance, studies have reported that subjective experiences, such as stress and childhood adversities, certain infections, and variable dose-dependent outcomes, including cannabis use, may have an impact during specific developmental stages of SCZ [14, 15]. However, these studies have focused on patients from the general population using a macro perspective, rarely including a unique population. In particular, most sample sources of WES studies are from the general population in China, of which samples from the Han people have been used to identify novel genetic susceptibility loci of SCZ [16]. Notably, relatively isolated populations will tend toward homogeneity in terms of genetic background and environmental exposure. For example, the isolated population of the Faroe Islands displays the mutation of glycogen storage disease III 4250 times more frequent than in outbred populations [17]. The results of independent samples precisely resolved the complex data caused by many interdependent environmental factors in other studies. Therefore, Tibet represents an important location that may provide a unique patient population to study the interaction between genes and the environment.

In this study, we completed screening and diagnosis of severe mental diseases in the Ngari prefecture, which is located in northwest Tibet, the highest average altitude in the world with a sparse population. In this survey, we visited the seven counties of the Nagri Prefecture, which has a population of approximately 0.1 million, and the total area is approximately 0.3 million km^2^. Our screening suggested that SCZ is the most common severe mental disorder in this area. We therefore performed WES to identify rare risk variants of SCZ in this area by investigating 47 individuals with SCZ and 53 controls from the isolated population. We also attempted to verify these findings in a follow-up Han sample of 279 SCZ patients and 95 healthy controls (HCs)

## Materials and methods

### Tibet subjects

Patients were included from seven counties in the Nagri Prefecture, whose diagnosis were made experienced psychiatrists from the Third People’s Hospital of Foshan based on all the material and records, according to the Diagnostic Criteria for Research (ICD-10), and the Diagnostic and Statistical Manual of Mental Disorders Fourth Edition (DSM-IV). HCs from the local community that were assessed as having no psychiatric record, were recruited by public advertising and included in this study.

### Han subjects

The Han participants included 279 patients with SCZ and 95 HCs, of which 99 patients and 45 HCs were from the Huangshan Second People’s Hospital while the rest were from the Third People’s Hospital of Foshan. All patients with SCZ were diagnosed by experienced psychiatrists according to the ICD-10 and a Structured Clinical Interview using the DSM-IV. The HCs consisted of local volunteers recruited through public advertising.

All the participants or their relatives signed an informed consent form. The authors assert that all procedures contributing to this work comply with the ethical standards of the relevant national and institutional committees on human experimentation and with the Helsinki Declaration of 1975, as revised in 2008. All procedures involving human subjects/patients were approved by the Biological and medical ethics committee, Minzu University of China.

### Sequence processing of Tibetan subjects

#### Library and sequencing

The library preparation was performed according to the manufacturer’s instructions, and the exome was captured using Agilent Sure Select version 3 (Agilent Technologies). The libraries were sequenced on an Illumina HiSeq2500 (Illumina).

#### Mapping

The sample reads were aligned to the genome (reference hg19) using BWA-MEM, converted to the BAM format, and indexed using SAM tools (version 0.1.18, https://samtools.github.io). The samples were realigned, marked for duplicates, and recalibrated using GATK as a pipeline manager.

### Sequence processing of Han subjects

#### Objective gene primer design

The primers were designed by the company (Novogene) using Primer 3 Online (http://frodo.wi.mit.edu /), Oligo software, and the National Center for Biotechnology Information (http://www.ncbi.nlm.nih.gov/).

#### Library preparation

Library preparation was performed in accordance with the manufacturer’s instructions. This mainly included PCR amplification, DNA purification, and library mixing.

#### High throughput sequencing (HTS)

The libraries were sequenced using an Illumina Hi-SNP (Illumina, Novogene, Beijing, China). The operation was performed in accordance with the standard SOP.

Variation detection and annotation. Variant type was determined using GATK Variant Annotator. Based on this, variant calls were grouped into single-nucleotide variants (SNVs) and insertion-deletion (indel). The values of SIFT, Polyphen2, and Polyphen2-HDIV were used to annotate missense mutations with additional predictions of potentially damaging consequences.

#### Association analysis

First, the variants were deeply filtered to select high-quality variations for association analysis. The filtering standard was as follows: this variant position had at least 95% of the samples reaching a depth of more than 10×. After deep filtering, correlation analysis was carried out by various methods according to different range values of MAF to divide the difference between the case and control. It was mainly divided into single variant association (SVA) and gene-wise association (GWA). For SVA, we used PLINK to perform case/control association analysis while ignoring the variants of Hawin imbalance (*P* < 1e−5). For GWA, we used EPACTS software to perform gene-level association analysis of variants grouped by gene.

### Metascape analysis of the variant target genes

Metascape pathway enrichment analysis was performed to identify new potential pathogenic and rare damaging variants. The TargetScan database (version 6.2) was used to predict target gene variants, and the threshold of TargetScan context+ scores was set to −0.20.

## Results

### Tibet sample sequencing

**Fig. 1:**
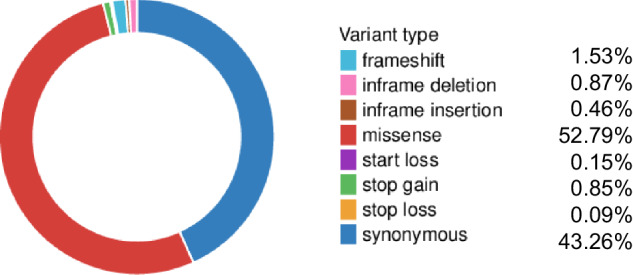
The proportion of sequenced variant types. There were eight types of variants, with missense being the most common and stop loss being the least prevalent.

WES analysis of the 47 SCZ cases and 53 controls was performed at an average depth of 122.98. After comparing the sequencing reads to the human reference genome using BMA-MEM and filtering out low-quality variations using GATK, a total of 213,097 variants were identified. These included 199,521 SNVs and 13,558 Indels. Overall, 27,644 of the called variants were novel and not present in the Single Nucleotide Polymorphism Database (dbSNP). The variant types are shown in Fig. 1. All known pathogenic mutations in SCZ-related genes were obtained by searching the Human Gene Mutation Database (HGMD), and the distribution of these mutations in the samples was determined. The results showed that *12:46244485:A/G* (*ARID2*) and *22:18905934:A/G* (*PRODH*) may be pathogenic variants of SCZ.

This study was designed to target rare risk variants that might have increased in frequency in the isolated plateau population. Consequently, we included only variants at frequencies lower than 0.05 in the genomes data. In addition, due to the small sample size, we limited the analysis to those variants carried by two or more cases but not by controls, which were low frequency, harmful, and conserved sites. Thus, these variants may be pathogenic. This filtering strategy revealed 275 new potentially pathogenic variants (Supplementary Table), these genes included *MAP2*, *IL6R*, *SHANK1* and *BAI2*.

For variant burden analysis, we counted the number of cases and controls carrying these variants at each low-frequency and harmful (frameshift, stop gain, spreading, or sift/polyphen2 predicted as harmful missense variant) gene. Among them, the number of cases was significantly greater than the number of controls, which may indicate potential pathogenic genes. Subsequently, 27 rare, damaging variants were identified (Table 1).

**Table 1:** Rare damaging variants from the Tibet samples.

Gene	Case number	Control number	*P* value (Fisher’s exact test, alternative = “greater”)
NPIPA3	13/46	2/51	0.000920854
MUC12	39/46	30/51	0.004284524
AGAP7	6/46	0/51	0.009478932
NPIPB5	8/46	1/51	0.01008246
SPATA31A7	11/46	3/51	0.011902438
POTEI	44/46	40/51	0.012244578
FCGBP	19/46	10/51	0.017244393
C10orf120	7/46	1/51	0.020693933
ARHGEF11	5/46	0/51	0.021269799
C10orf112	5/46	0/51	0.021269799
ZNF544	9/46	3/51	0.0404848
GOLGA6L3	6/46	1/51	0.041353144
SSH1	6/46	1/51	0.041353144
TPSB2	6/46	1/51	0.041353144
WASH2P	26/46	19/51	0.044718187
ANKRD30A	4/46	0/51	0.047097413
C11orf80	4/46	0/51	0.047097413
CASP5	4/46	0/51	0.047097413
GPRASP2	4/46	0/51	0.047097413
LAMA1	4/46	0/51	0.047097413
MAP2	4/46	0/51	0.047097413
NCR2	4/46	0/51	0.047097413
SELPLG	4/46	0/51	0.047097413
SFN	4/46	0/51	0.047097413
SOGA2	4/46	0/51	0.047097413
SOX18	4/46	0/51	0.047097413
SYCP2	4/46	0/51	0.047097413

### Metascape analysis of new variants

**Fig. 2:**
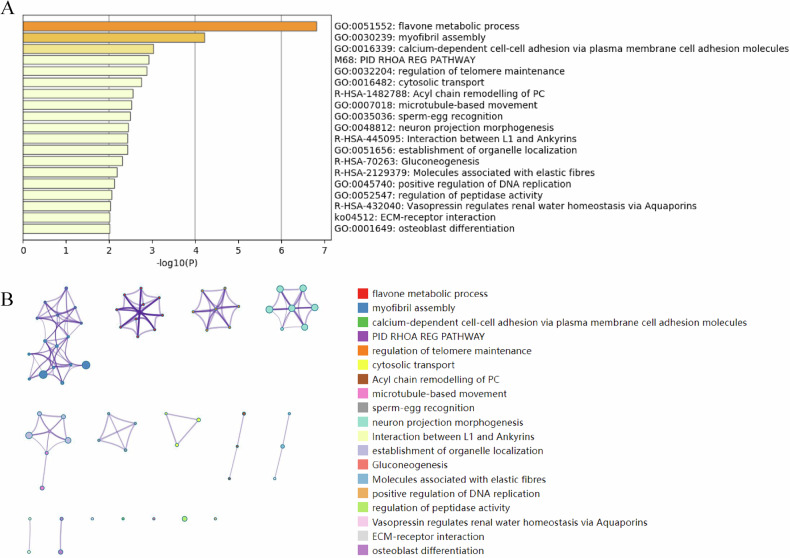
Bioinformatics analysis of potential risk genes in schizophrenia. **A** The top 20 Metascape enrichment clusters of potential risk genes in the isolated population. **B** Metascape enrichment network analysis depicting the intracluster and intercluster similarities of enriched terms for the potential risk genes.

To comprehend the potential function of these variants in SCZ, Metascape enrichment analysis was performed for the corresponding genes, including new potential pathogenic variants and rare damaging variants. The top five Metascape enrichment pathways included the flavone metabolic process, myofibril assembly, calcium-dependent cell-cell adhesion via plasma membrane cell adhesion molecules, the PID RHOA REG PATHWAY, and regulation of telomere maintenance (Fig. 2A). In addition, the enrichment networks of the top enrichment clusters were used to analyze intracluster and intercluster relatedness (Fig. 2B). The analyses suggested that high intracluster similarities drove the formation of tight local complexes and a substantial proportion of clusters were bridged through subterms with similarities.

### Association analysis

We first filtered the variation deeply and selected high-quality variations for the association analysis. After deep filtering, association analysis was carried out by various methods according to the different range values of MAF to determine the difference between cases and controls. For SVA, 86,574 common variants (MAF ≥ 0.05) and 54,503 low frequency variants (0.01 ≤ MAF < 0.05) were identified after filtering. Ignoring the Hawin imbalance, 85,509 common variants and 54 503 low frequency variants remained. The analysis of common variants showed a significant association (*P* < 0.05) of 4495 reference genes in the trend test, 4500 genes under the Allelic Model, 947 genes under the Dominant Model, 443 genes under the Recessive Model, and 685 genes under the Genotypic Model.

**Fig. 3:**
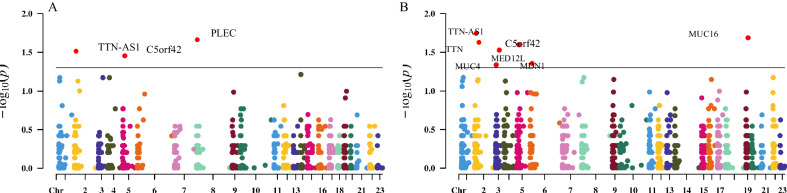
Manhattan plots of the genome-wide association studies with the rare variants. **A** Three genes exhibited a significant difference in damage stop-gain-frameshift varients. **B** Seven genes exhibited a significant difference in nonsynonymous variants.

To perform GWA on the rare variants, we divided variants with predicted significance related to SCZ into different groups for analysis. *C5orf42* was the only significant gene in both groups (*P* < 0.05), regardless of whether it was a damage stopgain-frameshift (Fig. 3A) or nonsynonymous variant (Fig. 3B).

### Verification of the variants related to SCZ in Han subjects

To explore whether the general population has the same mutation trend, we verified 47 variants (Table 2) related to SCZ filtering from the new potential pathogenic variants and rare damaging variants in the Chinese Han population. The average depth reached 2851 in these test samples using BWA software. However, the results showed that only *BAI2* variants appeared in the case group, with one in the Han population and two in the Tibetan population. The unique SCZ risk variant signature in Ngari Prefecture may be due to the fact that these people live under extreme environmental conditions, and they are a genetically homogeneous population due to geographic isolation.

**Table 2:** The verification of 47 genes in the Han population.

Gene	Consequence	rs	VID	hgvs	Function
APBA3	missense_variant	rs146584090	19:3751091:T/C	NM_004886.3:c.1661 A > G(p.His554Arg)	It is an adapter protein that interacts with the Alzheimer’s disease amyloid precursor protein. This gene product is believed to be involved in signal transduction processes. This gene is a candidate gene for Alzheimer’s disease.
ARHGAP39	missense_variant	.	8:145755853:C/T	NM_025251.1:c.3298 G > A(p.Val1100Met)	Predicted to enable GTPase activator activity. Involved in postsynapse organization. Is active in glutamatergic synapse.
ARHGEF11	missense_variant	.	1:156906736:G/C	NM_014784.3:c.4382 C > G(p.Thr1461Ser)	The encoded protein may form a complex with G proteins and stimulate Rho-dependent signals. A similar protein in rat interacts with glutamate transporter EAAT4 and modulates its glutamate transport activity.
BAI2	missense_variant	rs200836738	1:32222059:G/A	NM_001703.2:c.379 C > T(p.Arg127Trp)	The encoded protein is a brain-specific inhibitor of angiogenesis. The mature peptide may be further cleaved into additional products (PMID:20367554). Alternative splicing results in multiple transcript variants.
C14orf177	missense_variant	.	14:99183438:T/C	NM_182560.2:c.205 T > C(p.Cys69Arg)	NA
CDH10	missense_variant	.	5:24487935:G/C	NM_006727.3:c.2204 C > G(p.Thr735Ser)	This gene encodes a type II classical cadherin of the cadherin superfamily. This particular cadherin is predominantly expressed in brain and is putatively involved in synaptic adhesions, axon outgrowth and guidance.
CLTCL1	inframe_deletion	rs782037820	22:19175545:TTG/-	NM_007098.3:c.4380_4382delCAA(p.Asn1460del)	This gene is a member of the clathrin heavy chain family and encodes a major protein of the polyhedral coat of coated pits and vesicles.
CPLX4	missense_variant	.	18:56985594:G/A	NM_181654.3:c.101 C > T(p.Pro34Leu)	This gene likely encodes a member of the complexin family. The encoded protein may be involved in synaptic vesicle exocytosis.
DNAH5	missense_variant	rs200983202	5:13759047:C/G	NM_001369.2:c.10327 G > C(p.Asp3443His)	This gene encodes a dynein protein.This protein is an axonemal heavy chain dynein. It functions as a force-generating protein with ATPase activity, whereby the release of ADP is thought to produce the force-producing power stroke.
DPPA4	missense_variant	.	3:109047930:T/C	NM_018189.3:c.685 A > G(p.Arg229Gly)	This gene encodes a nuclear factor that is involved in the maintenance of pluripotency in stem cells and essential for embryogenesis.
ERC1	missense_variant	.	12:1291147:A/T	NM_178039.2:c.1848A>T(p.Gln616His)	The protein encoded by this gene is a member of a family of RIM-binding proteins. RIMs are active zone proteins that regulate neurotransmitter release.
FUBP1	missense_variant	.	1:78430904:C/A	NM_003902.3:c.485 G > T(p.Arg162Leu)	The protein encoded by this gene is a single stranded DNA-binding protein that binds to multiple DNA elements. Aberrant expression of this gene has been found in malignant tissues, and this gene is important to neural system and lung development.
GEMIN7,ZNF296	missense_variant	.	19:45579582:G/A	NM_145288.1:c.50 C > T(p.Pro17Leu)	The protein encoded by this gene is a component of the core SMN complex, which is required for pre-mRNA splicing in the nucleus.
IL21R-AS1,IL21R	missense_variant	.	16:27460434:G/A	NM_181079.4:c.1513 G > A(p.Ala505Thr)	The protein encoded by this gene is a cytokine receptor for interleukin 21. This receptor transduces the growth promoting signal of IL21, and is important for the proliferation and differentiation of T cells, B cells, and natural killer (NK) cells.
IL6R	missense_variant	rs780683821	1:154437680:G/A	NM_000565.3:c.1231 G > A(p.Gly411Arg)	This gene encodes a subunit of the interleukin 6 (IL6) receptor complex. Interleukin 6 is a potent pleiotropic cytokine that regulates cell growth and differentiation and plays an important role in the immune response.
KCNAB1	missense_variant	.	3:155861100:C/T	NM_003471.3:c.133 C > T(p.Pro45Ser)	This gene encodes a member of the potassium channel, voltage-gated, shaker-related subfamily. Their diverse functions include regulating neurotransmitter release, heart rate, insulin secretion, neuronal excitability, epithelial electrolyte transport, smooth muscle contraction, and cell volume.
KIF17	missense_variant	rs200844482	1:21009232:C/T	NM_020816.2:c.2377 G > A(p.Gly793Arg)	Predicted to enable microtubule binding activity and plus-end-directed microtubule motor activity. Predicted to be involved in anterograde dendritic transport of neurotransmitter receptor complex and cell projection organization. Predicted to act upstream of or within microtubule-based process; protein-containing complex localization; and vesicle-mediated transport.
KNDC1	missense_variant	rs985546165	10:135009249:G/A	NM_152643.6:c.1658 G > A(p.Arg553His)	The protein encoded by this gene is a Ras guanine nucleotide exchange factor that appears to negatively regulate dendritic growth in the brain.
MAP2	missense_variant	.	2:210574665:A/G	NM_002374.3:c.4760 A > G(p.Lys1587Arg)	The proteins of this family are thought to be involved in microtubule assembly, which is an essential step in neurogenesis. The products of similar genes in rat and mouse are neuron-specific cytoskeletal proteins that are enriched in dentrites, implicating a role in determining and stabilizing dentritic shape during neuron development.
NPTX2	missense_variant	rs377548219	7:98256538:C/T	NM_002523.2:c.950 C > T(p.Thr317Met)	This gene encodes a member of the family of neuronal petraxins, synaptic proteins that are related to C-reactive protein. This protein is involved in excitatory synapse formation.
PGAM2	missense_variant	rs770622502	7:44104852:C/T	NM_000290.3:c.277 G > A(p.Gly93Arg)	This gene encodes muscle-specific Phosphoglycerate mutase subunit.
PLA2G4B,JMJD7-PLA2G4B	missense_variant	.	15:42138538:C/G	NM_005090.3:c.2431 C > G(p.His811Asp)	This gene encodes a member of the cytosolic phospholipase A2 protein family.
PTCH1	missense_variant	.	9:98209454:G/A	NM_000264.3:c.4084 C > T(p.Pro1362Ser)	This gene encodes a member of the patched family of proteins and a component of the hedgehog signaling pathway. Hedgehog signaling is important in embryonic development and tumorigenesis
SCN11A	missense_variant	.	3:38921541:G/A	NM_014139.2:c.3293 C > T(p.Thr1098Ile)	This gene encodes one member of the sodium channel alpha subunit gene family, and is highly expressed in nociceptive neurons of dorsal root ganglia and trigeminal ganglia.
SH3TC2	missense_variant	rs760656119	5:148392170:C/T	NM_024577.3:c.3181 G > A(p.Glu1061Lys)	The gene product has been proposed to be an adapter or docking molecule. Mutations in this gene result in autosomal recessive Charcot-Marie-Tooth disease type 4 C, a childhood-onset neurodegenerative disease characterized by demyelination of motor and sensory neurons.
SHANK1	missense_variant	rs577804387	19:51172180:G/A	NM_016148.2:c.3037 C > T(p.Pro1013Ser)	This gene encodes a member of the SHANK (SH3 domain and ankyrin repeat containing) family of proteins. Members of this family act as scaffold proteins that are required for the development and function of neuronal synapses. Deletions in this gene may be associated with autism spectrum disorder in males.
SLC39A6	missense_variant	.	18:33702217:T/C	NM_012319.3:c.1157 A > G(p.His386Arg)	SLC39A6 belongs to a subfamily of proteins that show structural characteristics of zinc transporters
SORL1	missense_variant	.	11:121420769:G/A	NM_003105.5:c.2152 G > A(p.Val718Met)	The encoded preproprotein is proteolytically processed to generate the mature receptor, which likely plays roles in endocytosis and sorting.
SSH1	missense_variant	.	12:109182656:T/A	NM_018984.3:c.2258 A > T(p.Lys753Met)	The protein encoded by this gene belongs to the slingshot homolog (SSH) family of phosphatases, which regulate actin filament dynamics.
SYNJ1	missense_variant	.	21:34053882:C/A	NM_203446.2:c.1394 G > T(p.Arg465Leu)	This gene encodes a phosphoinositide phosphatase that regulates levels of membrane phosphatidylinositol-4,5-bisphosphate. As such, expression of this enzyme may affect synaptic transmission and membrane trafficking.
SYT8	splice_acceptor_variant	.	11:1857115:G/C	NM_138567.3:c.301–1 G > C(.)	This gene encodes a member of the synaptotagmin protein family. Synaptotagmins are membrane proteins that are important in neurotransmission and hormone secretion, both of which involve regulated exocytosis.
TAOK2	missense_variant	rs768000716	16:29998819:C/T	NM_001252043.1:c.2887 C > T(p.Arg963Trp)	This gene encodes a serine/threonine protein kinase that is involved in many different processes, including, cell signaling, microtubule organization and stability, and apoptosis.
TENM1	missense_variant	.	X:123695656:C/T	NM_014253.3:c.2299 G > A(p.Gly767Arg)	It is expressed in the neurons and may function as a cellular signal transducer.
TMEM132A	missense_variant	rs777345475	11:60696363:G/A	NM_017870.3:c.797 G > A(p.Arg266Gln)	This gene encodes a protein that is highly similar to the rat Grp78-binding protein (GBP).
TSEN34	missense_variant	.	19:54696153:G/A	NM_024075.3:c.674 G > A(p.Arg225Lys)	A mutation in this gene results in the neurological disorder pontocerebellar hypoplasia type 2.
TUFM	missense_variant	.	16:28856781:C/T	NM_003321.4:c.268 G > A(p.Ala90Thr)	This gene encodes a protein which participates in protein translation in mitochondria. Mutations in this gene have been associated with combined oxidative phosphorylation deficiency resulting in lactic acidosis and fatal encephalopathy.
YLPM1	missense_variant	.	14:75276681:C/T	NM_019589.2:c.5008 C > T(p.Pro1670Ser)	Enables RNA binding activity. Predicted to be involved in regulation of telomere maintenance. Predicted to act upstream of or within negative regulation of transcription by RNA polymerase II.
TRIO	missense_variant	.	5:14488193:T/C	NM_007118.2:c.7456 T > C(p.Trp2486Arg)	This gene encodes a large protein that functions as a GDP to GTP exchange factor. This protein promotes the reorganization of the actin cytoskeleton, thereby playing a role in cell migration and growth.
RAB41	missense_variant	.	X:69502652:G/C	NM_001032726.2:c.181 G > C(p.Ala61Pro)	This gene encodes a small GTP-binding protein that belongs to the largest family within the Ras superfamily. These proteins function as regulators of membrane trafficking.
GPRASP2	missense_variant	rs770886846	X:101970390:C/T	NM_138437.5:c.593 C > T(p.Pro198Leu)	The encoded protein has been shown to be capable of interacting with several GPCRs, including the M1 muscarinic acetylcholine receptor and the calcitonin receptor.
INADL	inframe_deletion	.	1:62240913:TAA/-	NM_176877.2:c.757_759delAAT(p.Asn253del)	This gene encodes a protein with multiple PDZ domains. PDZ domains mediate protein-protein interactions, and proteins with multiple PDZ domains often organize multimeric complexes at the plasma membrane.
FOXP1	missense_variant	.	3:71102805:C/A	NM_032682.5:c.402 G > T(p.Gln134His)	This gene belongs to subfamily P of the forkhead box (FOX) transcription factor family. Forkhead box transcription factors play important roles in the regulation of tissue- and cell type-specific gene transcription during both development and adulthood.
NLRC5	missense_variant	rs1053181583	16:57060387:C/T	NM_032206.4:c.1532 C > T(p.Ala511Val)	This gene plays a role in cytokine response and antiviral immunity through its inhibition of NF-kappa-B activation and negative regulation of type I interferon signaling pathways.
LPCAT2	stop_gained	rs144432562	16:55575825:C/T	XM_005256006.1:c.928 C > T(p.Arg310Ter)	The encoded protein may function in membrane biogenesis and production of platelet-activating factor in inflammatory cells.
ABCA10	missense_variant	.	17:67211999:A/G	NM_080282.3:c.815 T > C(p.Leu272Ser)	NA
SUCLG2	missense_variant	.	3:67459404:G/C	XM_005264773.1:c.1117 C > G(p.His373Asp)	This gene encodes a GTP-specific beta subunit of succinyl-CoA synthetase. Succinyl-CoA synthetase catalyzes the reversible reaction involving the formation of succinyl-CoA and succinate.
PIK3C2A	missense_variant	.	11:17113578:C/T	NM_002645.2:c.4607 G > A(p.Arg1536His)	The protein encoded by this gene belongs to the phosphoinositide 3-kinase (PI3K) family. PI3-kinases play roles in signaling pathways involved in cell proliferation, oncogenic transformation, cell survival, cell migration, and intracellular protein trafficking.

## Discussion

Aiming to identify rare risk variants of SCZ, we attempted to take advantage of using a related isolated population, the Tibetan population from the Ngari Prefecture, as some of the variants that are very rare in outbred populations have been found to be highly consistent in loci or increased in frequency. In particular, this population lives at the highest average altitude in the world; therefore, the independence and particularity of this research is self-evident as these individuals live in a hypoxic environment due to the high altitude of the region. Notably, previous studies have reported that flavonoids could improve the injury caused by hypoxia [18, 19], which is consistent with our results that showed enriched genes in the flavone metabolic process, conforming to the characteristics of these populations in hypoxic environments. Flavone compounds have previously been exploited as potential antipsychotic targets. For example, one flavone compound was found to have favorable effects in alleviating SCZ-like symptoms because of its high affinity for dopamine D2 and D3, and serotonin 5-HT1A, 5-HT2A receptors [20]. Another flavone compound was found to inhibit SCZ symptoms by inhibiting the physiologically crucial enzyme, phosphodiesterase 1 [21]. Hypoxia during neurodevelopment is one of several environmental factors associated with an increased risk of SCZ. In fact, previous research has suggested that hypoxia may impair oligodendrocyte function and myelination during neurodevelopment, thus potentiating the emergence of neurological diseases, such as SCZ [22, 23]. Studies indicate that *DISC*, which increases rare nonsynonymous mutations in patients and impairs the differentiation of oligodendrocytes, may play a role in the pathogenesis of SCZ [24, 25]. Furthermore, roughly half of the SCZ candidate genes identified are linked to ischemia-hypoxia [26, 27], supporting the close correlation between the selected population and SCZ. Indeed, ischemia-hypoxia response genes in the brain overlap with a subset of SCZ genes; related to monogenic disorders of the nervous system and synaptic function identified in recent SCZ GWAS studies [28]. Our findings support the role of the flavone metabolic pathway in SCZ, providing a potential therapeutic basis for this disease and supporting the importance of hypoxia in the onset and/or development of SCZ. Additionally, this study could offer fresh insights into understanding the mechanisms of SCZ and other psychiatric diseases that share genetic risk factors [29].

Risk alleles identified in isolated populations may be extremely rare in other populations or not observed elsewhere, suggesting that these new rare variants may provide new insights into SCZ. Although the sample size was very limited and thus prone to yielding spurious findings, we identified single variants that have already been reported in The Human Gene Mutation Database (HGMD), suggesting that several candidate genes of SCZ found here are common in multiple populations. Among these single variants, *PRODH* is best known as a risk gene for SCZ [30, 31]. Previous research has reported *PRODH* may mediate functional genetic variations in the neostriatal-frontal circuits, resulting in increased a risk for SCZ [32]. Moreover, *PRODH* encodes a proline dehydrogenase enzyme that catalyzes the first step of proline catabolism and is most likely involved in neuromediator synthesis in the CNS, especially in the hippocampus, which is known to be one of the brain structures most affected in SCZ [33]. Taken together, our findings provide further support for the role of this gene in susceptibility to SCZ.

Subsequently, 275 variants were revealed to have new potential pathogenicity, and 27 variants were revealed to cause rare damage via effective filtering. Among these variants, the *MAP2* missense variant is intriguing, although this variant has not been detected in the Han population. *MAP2* encodes a protein that belongs to the microtubule-associated protein family. Previous research has shown an association between *MAP6* and SCZ [34]. Proteins of this family are thought to be involved in microtubule assembly, which is an essential step in neurogenesis. Reduced neurogenesis marker expression is associated with polygenic risk in SCZ [35]. Moreover, decreased adult neurogenesis in the hippocampus of model mice has been found to be associated with the pathology of SCZ [36]. On the other hand, aberrant *MAP2* phosphorylation may underlie the profound reductions in MAP2-IR observed as a “molecular hallmark” of SCZ observed postmortem [37, 38], suggesting that *MAP2* could have direct consequences on neuronal structure and function in SCZ. Our findings support the role of this gene in susceptibility to SCZ and provide a good genetic basis for SCZ under hypoxic condition.

Notably, our association analysis showed *C5orf42* from both damage-stop-gain frameshift variants and nonsynonymous variants. *C5orf42* is also known as ciliogenesis and planar polarity effector 1 (*CPLANE1*). The protein encoded by this gene has putative coiled-coil domains and may be a transmembrane protein. In fact, the top-ranked psychosis-associated differentially methylated position (cg23933044), located in the promoter of the *C5ORF42* gene, was hypomethylated in post-mortem prefrontal cortex brain tissue from SCZ patients compared to unaffected controls [39]. Another genome-wide analysis showed that several hypomethylated genes were significantly enriched in the cerebral cortex and functionally enriched in nervous system development in SCZ [40]. Our findings support a potential role for this gene and connect the importance of methylation and SCZ, providing a basis for functional studies that reveal new epigenetic therapies.

Nevertheless, findings using isolated populations may not necessarily generalize to other populations making replication difficult. Therefore, we selected 47 new variants identified among the isolated population for verification in a general population, and only one risk gene emerged: *BAI2*. Notably, its family member, BAI3, has already been reported to be correlated with psychiatric disorders [41]. This gene is predominantly expressed in the brain, and while its physiological ligands and functions remain unclear, emotional behaviors were found to be modulated by *BAI2*, which connects with the main mediators of signal transduction, G protein-coupled receptors, in the central nervous system [42]. Interestingly while identified missense variants in both the isolated and general populations, they were in different loci, providing a novel and potential genetic mechanism of SCZ as well as revealing the importance of the *BAI2* gene in SCZ, although its functions and effects on the disorder remain unclear. Overall, our findings revealed novel variants across numerous genes in an isolated population, although replications of these genes in the general population were rare. This might provide opportunities to further investigate the pathogenesis regulated by different genes under extreme conditions. Indeed, investigating mutations in brain cells in SCZ is crucial, as brain damage occurring during the embryonic stage—which is later than the damage leading to neurodevelopmental disorders—could contribute to the development of schizophrenia during maturation and adulthood. Consequently, the analysis of somatic mutations may emerge as a promising approach in future research [43].

In summary, our results support both existing findings in the literature on SCZ, as well as new risk genes in the disease etiology. In particular, we identified rare variants that may directly lead to the underlying biology of SCZ under hypoxic conditions. Importantly, potential new risk variants could not be verified in the Chinese Han population, which suggests that SCZ patients living at high altitudes may have a unique risk gene signature.

### Supplementary information

Supplemental table

## Data Availability

Data available on request from the authors.
