# Correction: Whole-exome sequencing of individuals from an isolated population under extreme conditions implicates rare risk variants of schizophrenia

**DOI:** 10.1038/s41398-024-03023-6

**Published:** 2024-07-16

**Authors:** Lei Chen, Yang Du, Yang Hu, Xue-Song Li, Yuewen Chen, Yong Cheng

**Affiliations:** 1 Key Laboratory of Ethnomedicine of Ministry of Education, Center on Translational Neuroscience, School of Pharmacy, Minzu University of China, Beijing, China; 2 College of Life and Environmental Sciences, Minzu University of China, Beijing, China; 3 The Third People’s Hospital of Foshan, Foshan, China; 4 Chinese Academy of Sciences Key Laboratory of Brain Connectome and Manipulation, Shenzhen Key Laboratory of Translational Research for Brain Diseases, The Brain Cognition and Brain Disease Institute, Shenzhen Institute of Advanced Technology, Chinese Academy of Sciences; Shenzhen–Hong Kong Institute of Brain Science–Shenzhen Fundamental Research Institutions, Shenzhen, Guangdong 518055 China; 5 Guangdong Provincial Key Laboratory of Brain Science, Disease and Drug Development, HKUST Shenzhen Research Institute, Shenzhen, Guangdong 518057 China; 6 Institute of National Security, Minzu University of China, Beijing, China

**Keywords:** Schizophrenia, Genomics

Correction to: *Translational Psychiatry* 10.1038/s41398-024-02984-y, published online 29 June 2024

In the original version of this article Yuewen Chen has been omitted as corresponding author.

The original article has been corrected.

