# Supplementary material for: Whole-exome sequencing of individuals from an isolated population under extreme conditions implicates rare risk variants of schizophrenia
Source: Transl Psychiatry. 2024 Jun 29;14:267. doi: 10.1038/s41398-024-02984-y (PMC11217384; doi:10.1038/s41398-024-02984-y)
Supplement: Supplementary file 1 — Supplemental table [file 41398_2024_2984_MOESM1_ESM.pdf]

VID	hgvs	Known pathogenic variant	rs	Allele cou	Case numb	Allele cou	Control nu	SIFT	Polyphen2	Polyphen2	Gene	Consequence	MAX_AF	GERP++	phastCons	phastCons	phastCons	phyloP46w	phyloP46w	phyloP100	Note
X:153285086:G/C	XM_005274668.1:c.178C>G(p.Leu60Val)	no	.	5/92	3/46	0/102	0/51	D	.	.	IRAK1	missense_variant	0	-0.287	0.068	0.002	0	0.368	-0.267	-0.645	
4:74453619:T/C	NM_177532.4:c.320A>G(p.Tyr107Cys)	no	.	4/92	4/46	0/102	0/51	D	D	D	RASSF6	missense_variant	0	5.7	0.579	0.808	1	0.477	2.178	5.814	
4:191003060:C/G	NM_001177376.2:c.788C>G(p.Pro263Arg)	no	.	4/6	2/3	0/8	0/4	D	.	.	DUX4L4	missense_variant	0	0	0.001	0.001	0.011	-1.04	-0.972	0.554	
11:66610464:C/A	NM_024650.3:c.1892C>A(p.Pro631His)	no	.	4/92	4/46	0/102	0/51	D	D	P	C11orf80	missense_variant	0	1.44	0.153	0	0	0.591	0.082	0.66	
11:104871223:C/T	NM_004347.3:c.718-1G>A(.)	no	rs74729728	4/92	4/46	0/102	0/51	.	.	.	CASP5	splice_acceptor_variant	0.000061	3.91	0.01	0.127	1	0.205	1.909	4.428	
12:109182656:T/A	NM_018984.3:c.2258A>T(p.Lys753Met)	no	.	4/92	4/46	0/102	0/51	D	D	D	SSH1	missense_variant	0	4.05	0.931	0.734	0.863	0.533	0.918	1.187	
14:75276681:C/T	NM_019589.2:c.5008C>T(p.Pro1670Ser)	no	.	4/92	4/46	0/102	0/51	T	D	P	YLPM1	missense_variant	0	5.56	0.997	1	0.952	0.655	2.608	3.955	
20:62679821:C/G	NM_018419.2:c.853G>C(p.Gly285Arg)	no	rs75130170	4/92	4/46	0/102	0/51	D	P	P	SOX18	missense_variant	0	0.838	0.186	0.184	0.043	0.305	0.349	0.483	
22:20640729:-/TCC	XM_003403490.4:c.647_648insCAACGAGGA(p.Ala216_Ala217insAsnGluAs	no	.	4/86	2/43	0/88	0/44	.	.	.	LOC72946	inframe_insertion	0	.	.	.	.	.	.	.	
1:156906736:G/C	NM_014784.3:c.4382C>G(p.Thr1461Ser)	no	.	3/92	3/46	0/102	0/51	D	D	D	ARHGEF11	missense_variant	0	4.72	0.95	1	1	0.561	2.429	4.497	
1:161693220:G/A	NM_001002901.2:c.116G>A(p.Arg39Gln)	no	rs14195034	3/92	3/46	0/102	0/51	D	D	B	FCRLB	missense_variant	0.000061	3.91	0.773	0.526	1	0.655	0.787	3.231	
2:39107848:G/C	NM_001145450.1:c.-1+1G>C(.)	no	.	3/92	3/46	0/102	0/51	.	.	.	MORN2	splice_donor_variant	0	5.33	0.91	1	1	0.655	2.669	5.979	
2:89986796:-/GTG	NC_000002.11:IGKV2D-29:u_t_1.1:c.78_79insTGCAGAGTGTGATG(p.Thr27	no	rs77328225	3/92	3/46	0/102	0/51	.	.	.	IGKV2D-29	stop_gained	0.000064	.	.	.	.	.	.	.	
2:97403735:C/T	NM_001142292.1:c.257G>A(p.Arg86His)	no	rs78076810	3/92	3/46	0/102	0/51	D	D	D	LMAN2L	missense_variant	0.000086	3.59	0.991	1	1	-0.258	0.637	7.347	
2:206364706:A/G	NM_057177.6:c.2924A>G(p.Tyr975Cys)	no	rs76657562	3/92	3/46	0/102	0/51	D	D	D	PARD3B	missense_variant	0	4.43	0.022	0.853	1	0.46	1.052	4.715	
2:210574665:A/G	NM_002374.3:c.4760A>G(p.Lys1587Arg)	no	.	3/92	3/46	0/102	0/51	T	D	D	MAP2	missense_variant	0	4.27	0.011	0.975	1	-0.316	0.972	4.707	
3:15765993:C/T	NM_015199.3:c.589G>A(p.Ala197Thr)	no	.	3/92	3/46	0/102	0/51	D	D	D	ANKRD28	missense_variant	0	5.68	0.995	1	1	0.65	2.678	7.778	
4:139937277:-/CGC	NM_012118.3:c.155_163dupCGGCCTCGG(p.Ala52_Ser54dup)	no	.	3/92	3/46	0/102	0/51	.	.	.	CCRN4L	inframe_insertion	0	.	.	.	.	.	.	.	
6:31784989:C/G	NM_005345.5:c.1456C>G(p.Leu486Val)	no	.	3/92	3/46	0/100	0/50	D	D	D	HSPA1L,HS	missense_variant	0	3.54	0.99	1	0.956	0.423	2.003	2.07	
7:87514408:T/C	NM_006716.3:c.334T>C(p.Tyr112His)	no	.	3/92	3/46	0/102	0/51	T	P	P	DBF4	missense_variant	0	4.26	0.995	0.967	0.281	0.482	0.881	0.249	
7:99906624:G/A	NM_001004351.4:c.134G>A(p.Arg45His)	no	.	3/90	2/45	0/96	0/48	D	.	.	SPDYE3	missense_variant	0	0.19	0.001	0.001	0.988	0.299	0.294	0.766	
7:124499020:T/A	NM_015450.2:c.693A>T(p.Arg231Ser)	no	.	3/92	3/46	0/102	0/51	D	B	B	POT1	missense_variant	0	2.37	0.951	0.703	0.999	0.482	0.174	1.145	
8:145755853:C/T	NM_025251.1:c.3298G>A(p.Val1100Met)	no	.	3/92	3/46	0/102	0/51	T	D	D	ARHGAP39	missense_variant	0	4.02	0.865	0.94	0.001	-0.258	1.049	-0.115	
9:98209454:G/A	NM_000264.3:c.4084C>T(p.Pro1362Ser)	no	.	3/92	3/46	0/102	0/51	D	B	B	PTCH1	missense_variant	0	5.12	0.969	0.983	1	0.655	2.659	3.59	
10:100152257:C/T	NM_032709.2:c.994G>A(p.Glu332Lys)	no	.	3/92	3/46	0/102	0/51	D	P	P	PYROXD2	missense_variant	0	5	0.257	0.808	1	0.655	2.299	7.289	
11:587399:G/C	NM_020901.2:c.355G>C(p.Ala119Pro)	no	.	3/92	3/46	0/102	0/51	T	P	B	PHRF1	missense_variant	0	4.81	0.053	0.999	1	0.561	2.208	6.922	
11:118531292:A/G	NM_007180.2:c.1058T>C(p.Leu353Pro)	no	.	3/92	3/46	0/102	0/51	D	D	D	TREH	missense_variant	0	5.24	0.991	1	1	0.533	1.975	8.112	
11:123677046:C/T	NM_001005325.1:c.12G>A(p.Trp4Ter)	no	.	3/92	3/46	0/102	0/51	.	.	.	OR6M1	stop_gained	0	2.64	0.004	0.005	0	0.637	0.677	0.153	
12:6457283:C/T	NM_001038.5:c.1766G>A(p.Arg589Gln)	no	rs77529086	3/92	3/46	0/102	0/51	D	D	P	SCNN1A	missense_variant	0.000087	1.61	0.985	1	0.969	0.561	0.905	1.985	
12:109018001:G/C	NM_001206609.1:c.131C>G(p.Ala44Gly)	no	.	3/92	3/46	0/102	0/51	T	P	P	SELPLG	missense_variant	0	1.17	0.059	0.002	0.043	-0.31	0.326	1.023	
12:112037024:C/A	NM_002973.3:c.295G>T(p.Gly99Trp)	no	.	3/92	3/46	0/102	0/51	D	D	D	ATXN2	missense_variant	0	3.57	0.899	1	0.977	0.442	1.691	2.625	
14:99183438:T/C	NM_182560.2:c.205T>C(p.Cys69Arg)	no	.	3/92	3/46	0/102	0/51	D	B	B	C14orf177	missense_variant	0	-2.65	0.002	0	0	-0.25	-0.602	-0.247	
15:86123865:C/T	NM_006738.5:c.2566C>T(p.Leu856Phe)	no	.	3/92	3/46	0/102	0/51	D	D	D	AKAP13	missense_variant	0	5.68	0.009	0.009	0.009	0.655	2.673	2.41	
17:11603090:A/G	NM_001372.3:c.4915A>G(p.Met1639Val)	no	.	3/92	3/46	0/102	0/51	D	B	B	DNAH9	missense_variant	0	3.2	0.957	1	1	0.533	0.88	2.215	
17:79862997:C/G	NM_001184917.1:c.1057G>C(p.Asp353His)	no	.	3/92	2/46	0/102	0/51	D	D	D	PCYT2	missense_variant	0	3.83	0.313	0.947	0.99	0.561	2.497	2.868	
18:48723608:GGC	NM_016626.4:c.81_83delGCC(p.Pro31del)	no	rs55795386	3/90	3/45	0/98	0/49	.	.	.	MEX3C	inframe_deletion	0	.	.	.	.	.	.	.	
19:1789594:G/C	NM_138813.3:c.2611C>G(p.Arg871Gly)	no	.	3/92	3/46	0/102	0/51	T	P	B	ATP8B3	missense_variant	0	1.25	0.029	0.166	0.011	0.4	0.514	0.071	
19:45579582:G/A	NM_145288.1:c.50C>T(p.Pro17Leu)	no	.	3/92	3/46	0/102	0/51	D	P	B	GEMIN7,ZN	missense_variant	0	4.75	0.015	0.112	0.996	0.655	2.351	4.917	
20:58645887:C/T	NM_173644.1:c.305C>T(p.Ser102Phe)	no	.	3/92	3/46	0/102	0/51	D	B	B	C20orf197	missense_variant	0	-1.32	0.178	0	0.012	0.491	-0.296	0.679	
22:40662723:C/T	NM_001162501.1:c.2489C>T(p.Pro830Leu)	no	.	3/92	3/46	0/102	0/51	D	P	B	TNRC6B	missense_variant	0	3.09	0.013	0.994	0.434	-0.397	1.203	0.814	
X:18845418:G/A	NM_006240.2:c.1775G>A(p.Arg592His)	no	rs94664474	3/92	2/46	0/102	0/51	T	P	B	PPEF1	missense_variant	0	2.36	0.382	0.085	0.988	0.591	0.042	2.298	
X:69502652:G/C	NM_001032726.2:c.181G>C(p.Ala61Pro)	no	.	3/92	2/46	0/102	0/51	D	P	B	RAB41	missense_variant	0	-0.421	0.054	0	0.988	0.513	-0.368	1.517	
X:101970390:C/T	NM_138437.5:c.593C>T(p.Pro198Leu)	no	rs77088684	3/92	2/46	0/102	0/51	D	B	B	ARMCX5-G	missense_variant	0.000099	4.17	0.017	0.022	0	0.6	1.168	1.035	
X:106788974:A/G	XM_042978.8:c.305A>G(p.Lys102Arg)	no	.	3/92	3/46	0/102	0/51	D	.	.	FRMPD3	missense_variant	0	5	0.997	1	1	0.486	2.088	7.095	
X:123695656:C/T	NM_014253.3:c.2299G>A(p.Gly767Arg)	no	.	3/92	2/46	0/102	0/51	T	D	D	TENM1	missense_variant	0	5.39	0.964	1	1	0.594	2.376	7.775	
1:1216674:G/A	NM_001130413.3:c.74G>A(p.Arg25Lys)	no	.	2/92	2/46	0/102	0/51	D	B	B	SCNN1D	missense_variant	0	-3.31	0.003	0	0	-2.122	-3.774	-1.83	
1:1268667:G/A	NM_152228.1:c.1508G>A(p.Cys503Tyr)	no	.	2/92	2/46	0/102	0/51	D	D	D	TAS1R3	missense_variant	0	4.57	0.967	0.609	1	0.456	2.108	6.064	
1:6479171:G/T	NM_019089.4:c.284C>A(p.Ala95Glu)	no	.	2/92	2/46	0/102	0/51	T	P	B	HES2	missense_variant	0	1.6	0.839	0.99	0.015	0.462	0.453	0.728	
1:12726696:T/C	NM_001013630.1:c.1174T>C(p.Cys392Arg)	no	.	2/92	2/46	0/102	0/51	D	D	P	AADACL4	missense_variant	0	3.35	0.007	0.003	0.774	0.533	0.714	1.936	
1:19209851:C/G	NM_170726.2:c.525G>C(p.Lys175Asn)	no	.	2/92	2/46	0/102	0/51	T	P	B	ALDH4A1	missense_variant	0	3.42	0.344	0.986	1	0.491	1.247	1.704	
1:21009232:C/T	NM_020816.2:c.2377G>A(p.Gly793Arg)	no	rs20084448	2/92	2/46	0/102	0/51	T	D	D	KIF17	missense_variant	0.000096	5.76	0.976	0.999	0.999	0.563	2.724	4.246	
1:32222059:G/A	NM_001703.2:c.379C>T(p.Arg127Trp)	no	rs20083673	2/92	2/46	0/102	0/51	D	D	P	BAI2	missense_variant	0.000064	2.27	0.666	0.981	0.952	0.462	0.682	1.488	
1:55223958:G/A	NM_152268.3:c.877C>T(p.Pro293Ser)	no	.	2/92	2/46	0/102	0/51	T	P	B	PARS2	missense_variant	0	5.66	0.124	0.995	1	0.655	2.665	7.803	
1:62240913:TAA/-	NM_176877.2:c.757_759delAAT(p.Asn253del)	no	.	2/92	2/46	0/102	0/51	.	.	.	INADL	inframe_deletion	0	.	.	.	.	.	.	.	
1:75184998:A/C	NM_001130042.1:c.323T>G(p.Leu108Arg)	no	.	2/92	2/46	0/102	0/51	D	P	P	CRYZ	missense_variant	0	5.74	0.019	0.215	0.918	0.477	2.19	8.301	
1:78430904:C/A	NM_003902.3:c.485G>T(p.Arg162Leu)	no	.	2/92	2/46	0/102	0/51	D	B	B	FUBP1	missense_variant	0	5.59	0.994	1	1	0.65	2.784	7.689	
1:148015749:G/C	XM_005272988.1:c.882C>G(p.Asp294Glu)	no	.	2/92	2/46	0/102	0/51	T	D	D	NBPF14	missense_variant	0	0	0.197	0.197	0.995	0.064	0.064	-0.699	
1:154437680:G/A	NM_000565.3:c.1231G>A(p.Gly411Arg)	no	rs78068382	2/92	2/46	0/102	0/51	D	D	D	IL6R	missense_variant	0.000061	4.93	0.868	0.971	1	0.655	2.567	4.084	
1:155168254:G/C	NM_007112.4:c.2020C>G(p.Pro674Ala)	no	.	2/92	2/46	0/102	0/51	D	D	D	THBS3	missense_variant	0	5.06	0.98	1	1	0.462	2.631	8.01	
1:156228921:C/T	NM_015327.2:c.2317G>A(p.Gly773Ser)	no	.	2/92	2/46	0/102	0/51	D	D	D	SMG5	missense_variant	0	4.82	0.836	0.889	1	0.655	1.437	7.455	
1:156812810:T/C	NM_014215.2:c.3112A>G(p.Lys1038Glu)	no	.	2/92	2/46	0/102	0/51	D	B	B	NTRK1,INS	missense_variant	0	3.89	0.998	1	1	0.533	0.91	4.886	
1:156878799:C/T	NM_001080471.1:c.1382C>T(p.Pro461Leu)	no	.	2/92	2/46	0/102	0/51	D	D	D	PEAR1	missense_variant	0	4.65	0.651	0.166	0.998	0.563	2.387	4.373	
1:159752117:C/T	NM_017823.3:c.442C>T(p.Arg148Ter)	no	rs75335136	2/92	2/46	0/102	0/51	.	.	.	DUSP23	stop_gained	0	4.08	0.928	1	1	0.561	2.103	1.792	
1:167032899:G/A	NM_005814.1:c.491C>T(p.Ser164Leu)	no	.	2/92	2/46	0/102	0/51	D	D	D	GPA33	missense_variant	0	5.64	0.714	0.07	0.998	0.638	2.67	5.52	
1:197482061:TTAA	NM_001195215.1:c.1526_1538delAAAGGCCTCTTAA(p.Lys509ArgfsTer45)	no	.	2/92	2/46	0/102	0/51	.	.	.	DENND1B	frameshift_variant	0	.	.	.	.	.	.	.	
1:213178508:C/A	NM_144567.3:c.1001G>T(p.Ser334Ile)	no	rs77957615	2/92	2/46	0/102	0/51	D	P	P	ANGEL2	missense_variant	0.000086	3.71	0.993	0.999	1	0.65	0.686	2.239	
1:228432243:A/G	NM_001271223.2:c.3728A>G(p.Gln1243Arg)	no	.	2/92	2/46	0/102	0/51	T	D	P	OBSCN	missense_variant	0	1.26	0.23	0.02	1	0.369	0.067	5.355	
2:32738060:T/G	NM_016252.3:c.10407T>G(p.Ser3469Arg)	no	.	2/92	2/46	0/102	0/51	T	D	D	BIRC6	missense_variant	0	2.38	0.985	1	1	0.454	0.223	1.073	
2:44571692:C/A	NM_001171603.1:c.376G>T(p.Gly126Cys)	no	.	2/92	2/46	0/102	0/51	D	D	D	PREPL	missense_variant	0	4.9	0.996	1	1	0.65	2.729	1.537	
2:108475657:G/A	NM_182588.2:c.1364G>A(p.Arg455Gln)	no	.	2/92	2/46	0/102	0/51	T	D	D	RGPD4	missense_variant	0	2.6	0.452	0.998	1	0.152	1.299	4.818	
2:152362002:T/A	NM_001271208.1:c.23837A>T(p.His7946Leu)	no	.	2/92	2/46	0/102	0/51	D	D	D	NEB	missense_variant	0	5.44	0.989	1	1	0.529	2.199	6.928	
2:175436616:G/T	NM_001077269.1:c.917C>A(p.Ala306Asp)	no	.	2/92	2/46	0/102	0/51	D	P	P	WIPF1	missense_variant	0	4.53	0.073	0.969	1	0.43	2.07	4.703	
2:179613648:T/G	NM_133379.4:c.13479A>C(p.Lys4493Asn)	no	.	2/92	2/46	0/102	0/51	D	B	B	TTN	missense_variant	0	-3.11	0.432	0	0	0.46	-0.426	-0.04	
2:186667491:C/G	NM_173651.2:c.13725C>G(p.Ser4575Arg)	no	.	2/92	2/46	0/102	0/51	D	.	.	FSIP2	missense_variant	0	0.916	0.119	0	0	0.591	0.283	0.054	
2:203653742:T/G	NM_138468.4:c.1054A>C(p.Asn352His)	no	.	2/92	2/46	0/102	0/51	D	P	P	ICA1L	missense_variant	0	4.36	0.988	0.618	1	0.533	1.06	2.672	
2:231037574:A/-	NM_080424.2:c.1692delT(p.Val565TrpfsTer15)	no	.	2/92	2/46	0/102	0/51	.	.	.	SP110	frameshift_variant	0	-6.87	0.037	0	0	-0.327	-1.077	-1.805	
2:234591410:A/G	NM_019077.2:c.827A>G(p.Asn276Ser)	no	rs76577326	2/92	2/46	0/102	0/51	D	P	P	UGT1A8,U(	missense_variant	0.000061	1.17	0.103	0.967	1	0.397	0.541	2.714	
3:15270539:GACG	NM_014296.2:c.911_932delGACGTTTTAATAAGAAGTTAAT(p.Arg304IlefsTe	no	.	2/92	2/46	0/102	0/51	.	.	.	CAPN7	frameshift_variant	0	.	.	.	.	.	.	.	
3:38591993:G/A	NM_198056.2:c.5870C>T(p.Ser1957Phe)	no	.	2/92	2/46	0/102	0/51	D	P	B	SCN5A	missense_variant	0	4.95	0.423	0.975	0.998	0.655	2.573	2.931	
3:38921541:G/A	NM_014139.2:c.3293C>T(p.Thr1098Ile)	no	.	2/92	2/46	0/102	0/51	D	D	D	SCN11A	missense_variant	0	5.68	0.274	0.982	1	0.585	2.678	7.979	
3:44611851:G/A	NM_018651.2:c.1249G>A(p.Gly417Arg)	no	rs55032943	2/92	2/46	0/102	0/51	D	D	D	ZKSCAN7	missense_variant	0	4.35	0.991	0.982	1	0.65	2.267	5.379	
3:67459404:G/C	XM_005264773.1:c.1117C>G(p.His373Asp)	no	.	2/92	2/46	0/102	0/51	D	.	.	SUCLG2	missense_variant	0	0.111	0.087	0	0	0.563	0.015	-0.305	
3:71102805:C/A	NM_032682.5:c.402G>T(p.Gln134His)	no	.	2/92	2/46	0/102	0/51	D	D	D	FOXP1	missense_variant	0	3.73	0.994	1	1	-0.218	0.8	2.704	
3:109047930:T/C	NM_018189.3:c.685A>G(p.Arg229Gly)	no	.	2/92	2/46	0/102	0/51	D	P	B	DPPA4	missense_variant	0	-0.647	0.816	0.002	0	0.383	0.094	0.244	
3:112358138:G/T	NM_199511.1:c.615C>A(p.Ser205Arg)	no	rs75342847	2/92	2/46	0/102	0/51	D	P	P	CCDC80	missense_variant	0.000061	-2.48	0.926	0.887	0	-0.813	-0.643	-1.577	
3:155861100:C/T	NM_003471.3:c.133C>T(p.Pro45Ser)	no	.	2/92	2/46	0/102	0/51	T	P	B	KCNAB1	missense_variant	0	5.17	0.19	0.901	0.997	0.561	2.562	1.622	
3:186978571:G/A	NM_001031849.2:c.505C>T(p.Arg169Cys)	no	rs76801331	2/92	2/46	0/102	0/51	D	D	D	MASP1	missense_variant	0.000015	5.38	0.998	1	1	0.65	2.672	2.326	
3:195493563:C/T	NM_018406.6:c.13589G>A(p.Arg4530His)	no	rs37214114	2/92	2/46	0/102	0/51	D	D	D	MUC4	missense_variant	0	3.64	0.39	0.99	1	0.645	0.632	2.373	
4:1377704:G/A	NM_020894.2:c.2012G>A(p.Arg671His)	no	rs77637243	2/92	2/46	0/102	0/51	D	D	P	UVSSA	missense_variant	0.000016	4.98	0.34	0.237	1	0.499	2.466	8.197	
4:6865540:T/G	NM_014743.2:c.3431T>G(p.Phe1144Cys)	no	.	2/92	2/46	0/102	0/51	D	D	D	KIAA0232	missense_variant	0	4.9	0.731	0.852	1	0.533	1.97	7.592	
4:48575223:C/T	NM_015030.1:c.2884G>A(p.Ala962Thr)	no	rs75301888	2/92	2/46	0/102	0/51	D	D	D	FRYL	missense_variant	0.00003	5.8	0.979	1	1	0.655	2.737	7.405	
4:52709418:G/T	NM_001040402.1:c.10G>T(p.Asp4Tyr)	no	.	2/92	2/46	0/102	0/51	D	B	B	DCUN1D4	missense_variant	0	2.51	0.979	0.998	1	0.454	0.267	3.486	
4:73968123:C/T	NM_032217.3:c.4543G>A(p.Ala1515Thr)	no	.	2/92	2/46	0/102	0/51	T	P	B	ANKRD17	missense_variant	0	5.69	0.995	1	1	0.585	2.682	5.998	
4:77177353:G/T	NM_001242939.1:c.97G>T(p.Gly33Trp)	no	.	2/92	2/46	0/102	0/51	D	D	P	FAM47E-S	missense_variant	0	-10.5	0.001	0	0	-0.469	-1.553	-0.621	
4:85716040:A/G	NM_014991.4:c.3260T>C(p.Ile1087Thr)	no	.	2/92	2/46	0/102	0/51	D	B	B	WDFY3	missense_variant	0	5.44	0.704	0.998	1	0.533	2.194	6.964	
4:100128692:A/C	NM_001102470.1:c.875T>G(p.Val292Gly)	no	.	2/92	2/46	0/102	0/51	D	P	P	ADH6,LOC	missense_variant	0	0.852	0.001	0.003	0.063	-0.376	0.058	3.586	
4:119644717:C/T	NM_014822.2:c.3052G>A(p.Asp1018Asn)	no	rs53294836	2/92	2/46	0/102	0/51	D	D	D	SEC24D	missense_variant	0.000061	5.58	0.992	1	1	0.591	2.632	7.462	
4:187113081:A/G	NM_207352.3:c.104A>G(p.Gln35Arg)	no	rs77893638	2/92	2/46	0/102	0/51	T	P	B	CYP4V2	missense_variant	0.000097	0.19	0.145	0.123	0.483	0.358	-0.089	0.572	
5:484720:G/A	NM_004174.2:c.847C>T(p.Arg283Cys)	no	.	2/92	2/46	0/102	0/51	D	D	D	SLC9A3	missense_variant	0	4.16	0.976	0.998	1	0.561	1.856	4.126	
5:13759047:C/G	NM_001369.2:c.10327G>C(p.Asp3443His)	no	rs20098320	2/92	2/46	0/102	0/51	D	P	P	DNAH5	missense_variant	0	4.9	0.825	1	1	-0.188	1.437	7.722	
5:14488193:T/C	NM_007118.2:c.7456T>C(p.Trp2486Arg)	no	.	2/92	2/46	0/102	0/51	D	D	D	TRIO	missense_variant	0	4.99	0.996	1	1	0.454	1.873	4.979	
5:24487935:G/C	NM_006727.3:c.2204C>G(p.Thr735Ser)	no	.	2/92	2/46	0/102	0/51	D	P	P	CDH10	missense_variant	0	5.92	0.958	0.996	1	0.655	2.809	9.722	
5:56777648:G/A	NM_001017992.3:c.887C>T(p.Ala296Val)	no	.	2/92	2/46	0/102	0/51	.	P	P	ACTBL2	missense_variant	0	4.91	0.987	1	1	0.655	2.544	9.653	
5:112478942:A/G	NM_001085377.1:c.857T>C(p.Ile286Thr)	no	rs76496922	2/92	2/46	0/102	0/51	D	D	D	MCC	missense_variant	0.000061	5.66	0.996	1	1	0.533	2.285	6.956	
5:122337133:G/T	NM_014035.2:c.377+1G>T(.)	no	.	2/92	2/46	0/102	0/51	.	.	.	SNX24	splice_donor_variant	0	5.87	0.925	1	1	0.655	2.941	6.27	
5:127626525:A/T	NM_001999.3:c.6344T>A(p.Val2115Glu)	no	rs78068990	2/92	2/46	0/102	0/51	T	P	P	FBN2	missense_variant	0.000061	5.01	0.981	0.743	0.113	0.533	2.236	2.543	
5:131247544:AAAC	NM_001278059.1:c.664_667delGTTT(p.Val222LeufsTer51)	no	.	2/92	2/46	0/102	0/51	.	.	.	LOC72863	frameshift_variant	0	.	.	.	.	.	.	.	
5:140595449:T/C	NM_018933.2:c.1754T>C(p.Leu585Pro)	no	rs78278313	2/92	2/46	0/102	0/51	D	D	D	PCDHB13	missense_variant	0.000054	3.3	0.881	1	1	0.248	1.29	7.81	
5:148392170:C/T	NM_024577.3:c.3181G>A(p.Glu1061Lys)	no	rs76065611	2/92	2/46	0/102	0/51	D	D	P	SH3TC2	missense_variant	0.000061	5.87	0.856	0.922	1	0.655	2.78	7.445	
5:156712484:A/T	NM_001037333.1:c.113A>T(p.Tyr38Phe)	no	.	2/92	2/46	0/102	0/51	D	P	P	CYFIP2	missense_variant	0	5.61	0.993	1	1	0.533	2.254	8.798	
6:4087946:A/G	NM_001085401.2:c.71A>G(p.Glu24Gly)	no	.	2/92	2/46	0/102	0/51	D	P	P	C6orf201	missense_variant	0	3.08	0.294	0.314	1	0.46	0.759	1.345	
6:31695993:G/A	NM_013974.1:c.548C>T(p.Thr183Ile)	no	rs77535404	2/92	2/46	0/102	0/51	T	D	D	DDAH2	missense_variant	0.000016	4.6	0.968	0.971	1	0.655	2.391	4.99	
6:31974585:AGG/-	NR_040090.1:n.982_984delGGA(.)	no	rs10156688	2/92	2/46	0/102	0/51	.	.	.	CYP21A1P	splice_acceptor_variant	0	.	.	.	.	.	.	.	
6:41309544:C/G	NM_004828.3:c.407C>G(p.Thr136Arg)	no	.	2/92	2/46	0/102	0/51	T	P	P	NCR2	missense_variant	0	-2.18	0.297	0.017	0	-0.373	-0.689	-2.281	
6:41309793:C/T	NM_004828.3:c.550C>T(p.Arg184Cys)	no	rs77248847	2/92	2/46	0/102	0/51	T	D	B	NCR2	missense_variant	0.000045	-4.24	0.028	0	0	-0.373	-1.479	-0.87	
6:160968819:A/G	NM_005577.2:c.5304+2T>C(.)	no	rs75628661	2/92	2/46	0/102	0/51	.	.	.	LPA	splice_donor_variant	0.000045	2.5	0.152	0.213	1	0.163	1.163	6.3	
7:7483247:C/G	NM_001037763.2:c.1619G>C(p.Gly540Ala)	no	rs54397078	2/92	2/46	0/102	0/51	D	B	B	COL28A1	missense_variant	0.000061	2.05	0.785	0.355	0.443	0.655	0.339	0.507	
7:44104852:C/T	NM_000290.3:c.277G>A(p.Gly93Arg)	no	rs77062250	2/92	2/46	0/102	0/51	D	D	P	PGAM2	missense_variant	0.00003	5.93	0.993	0.989	1	0.65	2.833	7.652	
7:44796658:C/T	NM_031449.3:c.278C>T(p.Ala93Val)	no	.	2/92	2/46	0/102	0/51	T	P	B	ZMIZ2	missense_variant	0	4.16	0.964	0.853	0.997	0.655	1.343	3.049	
7:72436636:A/G	NM_198853.1:c.53T>C(p.Ile18Thr)	no	.	2/92	2/46	0/102	0/51	D	P	B	TRIM74	missense_variant	0	2.39	0.914	0.999	1	0.327	1.363	7.967	
7:92098446:T/C	NM_001130925.1:c.1250A>G(p.Lys417Arg)	no	.	2/92	2/46	0/102	0/51	D	.	.	ERVW-1	missense_variant	0	0.0465	0.355	0.354	0.621	0.113	0.115	0.147	
7:98256538:C/T	NM_002523.2:c.950C>T(p.Thr317Met)	no	rs37754821	2/92	2/46	0/102	0/51	D	D	D	NPTX2	missense_variant	0.000097	4.5	0.936	0.89	1	0.655	1.398	6.049	
7:99998699:C/T	NM_017984.4:c.1885G>A(p.Gly629Arg)	no	.	2/92	2/46	0/102	0/51	D	D	P	ZCWPW1	missense_variant	0	4.16	0	0.005	0.001	-0.136	1.508	1.149	
7:100284434:C/T	NM_022574.4:c.532G>A(p.Gly178Ser)	no	.	2/92	2/46	0/102	0/51	T	D	P	GIGYF1	missense_variant	0	4.96	0.85	0.99	0.126	0.563	2.571	1.521	
7:103969474:G/A	NM_199000.2:c.247G>A(p.Gly83Ser)	no	.	2/92	2/46	0/102	0/51	T	P	B	LHFPL3	missense_variant	0	4.17	0.998	1	1	0.558	1.116	5.347	
7:127338932:C/T	NM_014390.2:c.353C>T(p.Thr118Ile)	no	.	2/92	2/46	0/102	0/51	T	P	P	SND1	missense_variant	0	5.07	0.994	1	1	0.65	1.513	5.098	
7:138440483:C/A	NM_020632.2:c.767G>T(p.Arg256Leu)	no	rs77549606	2/92	2/46	0/102	0/51	D	D	D	ATP6V0A4	missense_variant	0.000061	6.17	0.22	1	1	0.655	2.941	7.327	
7:153749913:C/T	NM_130797.2:c.8C>T(p.Ser3Leu)	no	.	2/92	2/46	0/102	0/51	D	D	D	DPP6	missense_variant	0	3.31	0.718	1	1	0.549	1.562	4.242	
8:116723:A/T	NM_001005504.1:c.302T>A(p.Ile101Asn)	no	.	2/84	2/42	0/86	0/43	D	D	P	OR4F21	missense_variant	0	1.96	0.87	0.997	0.532	0.315	0.927	3.244	
8:8233844:G/T	NM_001080826.1:c.2075C>A(p.Ser692Tyr)	no	.	2/92	2/46	0/102	0/51	D	D	D	SGK223	missense_variant	0	5.42	0.611	0.895	1	0.655	2.725	6.474	
8:21891773:C/A	NM_182795.1:c.518C>A(p.Ala173Glu)	no	.	2/92	2/46	0/102	0/51	D	B	B	NPM2	missense_variant	0	-2.91	0.006	0	0	-0.291	-0.738	-0.345	
8:25246647:G/A	NM_024940.6:c.4172G>A(p.Arg1391Gln)	no	.	2/92	2/46	0/102	0/51	T	D	D	DOCK5	missense_variant	0	4.32	0.998	1	1	0.555	1.289	3.881	
8:71553193:G/A	NM_016027.2:c.685C>T(p.Arg229Cys)	no	rs75362393	2/92	2/46	0/102	0/51	D	D	B	LACTB2,LC	missense_variant	0.000017	1.7	0.894	0.004	0.496	0.632	0.335	1.896	
8:79598810:C/A	NM_016010.2:c.319C>A(p.Leu107Ile)	no	.	2/92	2/46	0/102	0/51	D	D	D	ZC2HC1A	missense_variant	0	5.35	0.998	1	0.999	0.585	2.667	3.509	
8:144789991:G/A	NM_001162914.1:c.289C>T(p.Gln97Ter)	no	.	2/92	2/46	0/102	0/51	.	.	.	CCDC166	stop_gained	0	3.11	0.715	0.922	1	0.561	0.855	3.118	
9:4635369:T/-	XM_005251497.1:c.257delA(p.Asn86ThrfsTer12)	no	.	2/92	2/46	0/102	0/51	.	.	.	SPATA6L	frameshift_variant	0	5.31	0.967	1	1	0.46	2.014	3.501	
9:36657344:C/T	NM_014791.3:c.1160C>T(p.Thr387Ile)	no	.	2/92	2/46	0/102	0/51	D	P	B	MELK	missense_variant	0	4.11	0.11	0.974	0.999	0.46	2.568	2.858	
9:70918118:C/T	NM_199135.4:c.251C>T(p.Ser84Leu)	no	rs88000060	2/88	2/44	0/92	0/46	D	B	B	FOXD4L3	missense_variant	0	3.72	0.051	0.003	0.01	0.455	1.774	2.198	
9:84609521:G/A	NM_001001670.2:c.4136G>A(p.Ser1379Asn)	no	.	2/92	2/46	0/102	0/51	T	D	P	SPATA31D	missense_variant	0	-1.82	0.007	0	0	0.655	-0.351	-0.075	
9:89561475:G/C	NM_002048.2:c.220C>G(p.Pro74Ala)	no	.	2/92	2/46	0/102	0/51	D	P	B	GAS1	missense_variant	0	2.44	0.852	1	0.8	0.305	1.164	0.602	
9:128509802:G/T	NM_006195.5:c.70G>T(p.Gly24Cys)	no	.	2/92	2/46	0/102	0/51	D	P	P	PBX3	missense_variant	0	0.462	0.915	0.931	1	-0.229	0.102	6.611	
10:17813375:A/G	NM_001098844.2:c.325A>G(p.Thr109Ala)	no	.	2/92	2/46	0/102	0/51	T	D	P	TMEM236	missense_variant	0	5.45	0.202	0.659	0.861	0.454	2.077	1.864	
10:19641093:C/G	XM_005252651.1:c.4308C>G(p.Phe1436Leu)	no	.	2/92	2/46	0/102	0/51	D	.	.	C10orf112	missense_variant	0	3.53	0.977	1	0.476	0.65	0.814	-0.046	
10:24873720:G/A	NM_020824.3:c.5498C>T(p.Ser1833Leu)	no	.	2/92	2/46	0/102	0/51	D	P	B	ARHGAP21	missense_variant	0	4.62	0.003	0.018	1	-0.15	1.311	9.555	
10:48388365:T/C	NM_002900.2:c.2513A>G(p.Lys838Arg)	no	.	2/92	2/46	0/102	0/51	D	D	D	RBP3	missense_variant	0	5.35	0.877	1	1	0.459	2.044	7.49	
10:51465786:A/G	NM_001077685.2:c.670T>C(p.Ser224Pro)	no	.	2/92	2/46	0/102	0/51	D	D	P	TIMM23B,A	missense_variant	0	0	0.007	0.007	1	0.147	0.149	4.4	
10:73521633:C/T	NM_022153.1:c.233G>A(p.Gly78Asp)	no	.	2/92	2/46	0/102	0/51	T	D	D	CDH23,C1(	missense_variant	0	4.46	0.949	0.114	0.034	0.655	2.767	3.268	
10:115380365:C/T	NM_006175.4:c.2767G>A(p.Glu923Lys)	no	.	2/92	2/46	0/102	0/51	D	D	D	NRAP	missense_variant	0	6.17	0.976	1	1	0.655	2.941	7.286	
10:134598468:G/C	NM_177400.2:c.786C>G(p.Asn262Lys)	no	.	2/92	2/46	0/102	0/51	D	P	B	NKX6-2	missense_variant	0	3.07	0.993	1	1	0.462	2.023	3.22	
10:135009249:G/A	NM_152643.6:c.1658G>A(p.Arg553His)	no	rs98554616	2/92	2/46	0/102	0/51	D	P	P	KNDC1	missense_variant	0	3.72	0.668	0.754	0.723	0.306	1.081	2.927	
11:1857115:G/C	NM_138567.3:c.301-1G>C(.)	no	.	2/92	2/46	0/102	0/51	.	.	.	SYT8	splice_acceptor_variant	0	2.07	0.247	0.886	1	0.305	0.589	4.333	
11:1955634:C/A	NM_006757.3:c.439C>A(p.Leu147Met)	no	.	2/92	2/46	0/102	0/51	D	D	D	TNNT3	missense_variant	0	4.66	0.883	0.959	0.732	0.313	2.314	1.425	
11:4592859:A/G	NM_144663.1:c.448T>C(p.Cys150Arg)	no	rs14852484	2/92	2/46	0/102	0/51	.	P	B	C11orf40	missense_variant	0.000047	-1.66	0.092	0	0	0.323	-0.692	-1.397	
11:6587942:C/T	NM_144666.2:c.11332C>T(p.Gln3778Ter)	no	.	2/92	2/46	0/102	0/51	.	.	.	DNHD1	stop_gained	0	0.217	0.131	0.17	0.121	-0.176	-0.035	0.072	
11:12399169:C/T	NM_018222.4:c.95C>T(p.Pro32Leu)	no	.	2/92	2/46	0/102	0/51	D	.	.	PARVA	missense_variant	0	2.94	0.421	0.97	0.816	-0.3	0.596	0.112	
11:17113578:C/T	NM_002645.2:c.4607G>A(p.Arg1536His)	no	.	2/92	2/46	0/102	0/51	D	D	D	PIK3C2A	missense_variant	0	5.85	0.997	1	1	0.655	2.772	7.399	
11:49075403:A/C	NM_001206631.1:c.1216T>G(p.Trp406Gly)	no	.	2/92	2/46	0/102	0/51	D	.	.	TRIM64C	missense_variant	0	-3.11	0.15	0	0	-1.392	-2.934	-2.101	
11:55944841:A/G	NM_001005492.1:c.748A>G(p.Ile250Val)	no	.	2/92	2/46	0/102	0/51	T	P	B	OR5J2	missense_variant	0	-4.96	0.003	0.064	0	-2.446	-0.732	-1.332	
11:60696363:G/A	NM_017870.3:c.797G>A(p.Arg266Gln)	no	rs77734547	2/92	2/46	0/102	0/51	D	D	D	TMEM132A	missense_variant	0.000087	3.83	0.958	0.995	1	0.555	1.143	2.389	
11:64112519:C/T	NM_032251.5:c.2506C>T(p.Arg836Trp)	no	rs76509171	2/92	2/46	0/102	0/51	D	D	D	CCDC88B	missense_variant	0	3.74	0.762	1	0.022	0.444	2.049	0.064	
11:93826793:-/TCT	NM_001098672.1:c.2422_2423insCTT(p.His807_Leu808insSer)	no	.	2/92	2/46	0/102	0/51	.	.	.	HEPHL1	inframe_insertion	0	.	.	.	.	.	.	.	
11:94040371:C/T	NM_001199206.1:c.389C>T(p.Pro130Leu)	no	rs77230499	2/92	2/46	0/102	0/51	D	D	P	FOLR4	missense_variant	0.000029	4.57	0.48	0.099	1	0.491	2.532	7.008	
11:117279715:C/G	NM_014956.4:c.3719C>G(p.Pro1240Arg)	no	.	2/92	2/46	0/102	0/51	D	P	P	CEP164	missense_variant	0	1.81	0.012	0.007	0.018	0.591	0.207	0.036	
11:120170375:C/T	NM_001244682.1:c.307C>T(p.Pro103Ser)	no	.	2/92	2/46	0/102	0/51	D	D	D	POU2F3	missense_variant	0	4.58	0.788	0.951	1	0.563	2.545	4.344	
11:121420769:G/A	NM_003105.5:c.2152G>A(p.Val718Met)	no	.	2/92	2/46	0/102	0/51	T	P	B	SORL1	missense_variant	0	5.38	0.994	1	1	0.655	2.518	4.912	
11:123909089:G/C	NM_001004463.1:c.620C>G(p.Ser207Trp)	no	.	2/92	2/46	0/102	0/51	D	D	D	OR10G7	missense_variant	0	0.249	0.362	0.639	0	0.557	-0.048	0.539	
11:124543604:T/C	NM_170601.4:c.1A>G(p.Met1?)	no	.	2/92	2/46	0/102	0/51	.	.	.	SIAE	start_lost	0	4.05	0.011	0.24	0.948	0.383	0.886	3.339	
12:1291147:A/T	NM_178039.2:c.1848A>T(p.Gln616His)	no	.	2/92	2/46	0/102	0/51	T	D	P	ERC1	missense_variant	0	1.36	0.98	0.999	1	0.533	-0.012	1.724	
12:6981483:T/C	NM_032641.3:c.583A>G(p.Lys195Glu)	no	.	2/92	2/46	0/102	0/51	D	D	P	SPSB2	missense_variant	0	3.69	0.671	1	1	0.46	1.669	5.97	
12:46322400:C/T	NM_004719.2:c.1084G>A(p.Ala362Thr)	no	.	2/92	2/46	0/102	0/51	D	D	P	SCAF11	missense_variant	0	6.07	0.995	1	1	0.585	2.89	1.944	
12:53817748:A/T	NM_020547.2:c.30A>T(p.Leu10Phe)	no	.	2/92	2/46	0/102	0/51	D	P	B	AMHR2	missense_variant	0	-0.96	0.992	0.831	0.824	0.533	0.018	0.312	
12:55945225:C/T	NM_001005494.1:c.215C>T(p.Thr72Ile)	no	rs75327893	2/92	2/46	0/102	0/51	D	B	B	OR6C4	missense_variant	0.000098	4.77	0.978	0.98	0.005	0.644	2.639	2.213	
12:56660924:C/T	NM_144576.3:c.22C>T(p.Arg8Trp)	no	rs56640032	2/92	2/46	0/102	0/51	D	D	B	COQ10A	missense_variant	0	2.91	0.286	0.915	1	0.561	0.898	1.586	

12:132512674:T/A	NM_015409.4:c.5222T>A(p.Leu1741Gln)	no	rs7645477[illegible]	2/92	2/46	0/102	0/51	D	P	B	EP400	missense_variant	0.000061	4.9	0.008	0.006	0.888	0.533	1.067	4.188	
13:97639638:A/G	NM_080818.3:c.376T>C(p.Cys126Arg)	no	.	2/92	2/46	0/102	0/51	D	D	D	OXGR1	missense_variant	0	5.56	0.996	0.999	1	0.533	2.118	5.952	
14:22788893:C/T	NC_000014.8:TRAV41:u_t_1.1:c.106C>T(p.Gln36Ter)	no	.	2/92	2/46	0/102	0/51	.	.	.	TRAV41	stop_gained	0	4.22	0.033	0.001	0	0.563	1.101	0.092	
14:22933290:G/-	NC_000014.8:TRDC:u_t_1.1:c.440delG(p.Asp148ThrfsTer?)	no	.	2/92	2/46	0/102	0/51	.	.	.	TRDC	frameshift_variant	0	4.29	0.421	1	1	0.655	1.156	0.926	
14:25102158:C/A	NM_004131.4:c.166G>T(p.Asp56Tyr)	no	.	2/92	2/46	0/102	0/51	D	B	B	GZMB	missense_variant	0	-1.72	0.129	0	0	-0.136	-0.192	-1.659	
14:31626072:T/C	NM_015382.2:c.1906A>G(p.Asn636Asp)	no	.	2/92	2/46	0/102	0/51	T	P	P	HECTD1	missense_variant	0	5.62	0.999	1	1	0.533	2.266	7.954	
14:92268643:G/A	NM_152332.4:c.424C>T(p.Arg142Ter)	no	.	2/92	2/46	0/102	0/51	.	.	.	TC2N	stop_gained	0	2.39	0.109	0.461	0.658	0.65	0.673	0.944	
14:92484048:C/T	NM_004239.3:c.635G>A(p.Cys212Tyr)	no	.	2/92	2/46	0/102	0/51	D	P	B	TRIP11	missense_variant	0	2.26	0.941	0.986	1	0.655	0.252	1.686	
15:42138538:C/G	NM_005090.3:c.2431C>G(p.His811Asp)	no	.	2/92	2/46	0/102	0/51	D	D	D	PLA2G4B,J	missense_variant	0	4.96	0.961	1	1	0.655	2.664	1.677	
15:42160738:G/C	NM_016642.3:c.5915C>G(p.Ser1972Trp)	no	.	2/92	2/46	0/102	0/51	D	D	D	SPTBN5	missense_variant	0	3.94	0.015	0.002	0.003	0.462	1.044	0.758	
15:42978232:G/A	NM_020759.2:c.4456G>A(p.Ala1486Thr)	no	.	2/92	2/46	0/102	0/51	D	.	.	STARD9	missense_variant	0	2.16	0.557	0.998	0.078	-0.251	0.375	0.284	
15:43658580:G/-	NM_152455.3:c.950delC(p.Pro317HisfsTer14)	no	.	2/92	2/46	0/102	0/51	.	.	.	ZSCAN29	frameshift_variant	0	4.85	0.984	1	0.963	0.563	2.506	1.256	
15:45394056:C/T	NM_014080.4:c.2786G>A(p.Arg929Gln)	no	rs75865847	2/92	2/46	0/102	0/51	T	P	B	DUOX2	missense_variant	0.000061	6.08	0.998	1	1	0.655	2.894	2.477	
15:65425866:G/A	NM_005707.1:c.254C>T(p.Pro85Leu)	no	.	2/92	2/46	0/102	0/51	D	P	B	PDCD7	missense_variant	0	2.76	0.781	0.766	1	0.313	1.534	4.921	
15:102029708:-/CA	NM_002570.3:c.163_165dupCTG(p.Leu55dup)	no	rs98950832	2/92	2/46	0/102	0/51	.	.	.	PCSK6	inframe_insertion	0	.	.	.	.	.	.	.	
16:4833464:C/T	NM_144605.4:c.725G>A(p.Arg242Gln)	no	rs99598697	2/92	2/46	0/102	0/51	D	D	P	12-Sep	missense_variant	0	3.79	0.367	0.995	1	0.462	1.233	7.495	
16:14820006:T/G	NM_001277323.1:c.909T>G(p.Asp303Glu)	no	.	2/88	2/44	0/100	0/50	D	P	P	LOC100652	missense_variant	0	0	0.039	0.039	0.137	0.076	0.077	0.077	
16:15474900:A/G	XM_003118702.2:c.5T>C(p.Val2Ala)	no	.	2/92	2/46	0/102	0/51	D	.	.	NPIPA5	missense_variant	0	0.62	0.106	0.137	0.955	0.092	0.54	1.531	
16:22155702:C/G	NM_173615.3:c.2727C>G(p.Ile909Met)	no	.	2/92	2/46	0/102	0/51	D	D	P	VWA3A	missense_variant	0	2.98	0.006	0.026	1	0.563	0.603	1.691	
16:27460434:G/A	NM_181079.4:c.1513G>A(p.Ala505Thr)	no	.	2/92	2/46	0/102	0/51	T	P	B	IL21R-AS1,	missense_variant	0	3.57	0.648	0.004	0.007	0.561	2.07	1.335	
16:28856781:C/T	NM_003321.4:c.268G>A(p.Ala90Thr)	no	.	2/92	2/46	0/102	0/51	D	D	D	TUFM	missense_variant	0	5.66	0.846	1	1	0.561	2.661	3.908	
16:29912608:C/A	NM_181718.3:c.316C>A(p.Leu106Ile)	no	.	2/92	2/46	0/102	0/51	D	P	B	ASPHD1	missense_variant	0	1.66	0.052	0.664	0.945	-1.598	0.185	1.215	
16:29998819:C/T	NM_001252043.1:c.2887C>T(p.Arg963Trp)	no	rs76800071	2/92	2/46	0/102	0/51	D	B	B	TAOK2	missense_variant	0	1.42	0.945	1	0.692	0.563	0.601	1.058	
16:55575825:C/T	XM_005256006.1:c.928C>T(p.Arg310Ter)	no	rs1444325[illegible]	2/92	2/46	0/102	0/51	.	.	.	LPCAT2	stop_gained	0	-1.87	0.482	0	0.001	-2.245	-0.324	-0.262	
16:57060387:C/T	NM_032206.4:c.1532C>T(p.Ala511Val)	no	rs1053181[illegible]	2/92	2/46	0/102	0/51	D	P	P	NLRC5	missense_variant	0	4.45	0.029	0.241	1	0.561	1.283	4.845	
16:67428759:C/G	NM_013304.2:c.1268G>C(p.Arg423Pro)	no	.	2/92	2/46	0/102	0/51	D	B	B	ZDHHC1	missense_variant	0	-3.97	0.535	0.022	0	-0.314	-0.547	-2.64	
16:74425595:G/A	XM_005256273.1:c.496G>A(p.Asp166Asn)	no	.	2/92	2/46	0/102	0/51	D	D	D	NPIPB15	missense_variant	0	0	0.03	0.03	0.017	0.074	0.073	0.075	
16:84690732:G/A	NM_024731.2:c.319G>A(p.Gly107Arg)	no	rs7531272[illegible]	2/92	2/46	0/102	0/51	D	D	P	KLHL36	missense_variant	0.00003	3.49	0.047	0.071	0.998	0.655	1.411	2.268	
16:87339436:T/C	NM_001195124.1:c.590A>G(p.Gln197Arg)	no	.	2/92	2/46	0/102	0/51	D	B	B	C16orf95	missense_variant	0	0.562	0.013	0.998	1	0.172	0.08	1.069	
17:4836474:C/G	NM_000173.5:c.575C>G(p.Thr192Ser)	no	rs74599231	2/92	2/46	0/102	0/51	D	B	P	GP1BA	missense_variant	0.000087	3.77	0.187	0.996	0.34	0.313	2.209	0.71	
17:4856394:T/C	NM_001976.4:c.230T>C(p.Leu77Pro)	no	.	2/92	2/46	0/102	0/51	D	D	D	ENO3	missense_variant	0	5.4	0.969	1	1	0.533	2.176	6.06	
17:6329947:G/A	NM_014336.3:c.772C>T(p.Arg258Trp)	no	rs5408759[illegible]	2/92	2/46	0/102	0/51	D	D	D	AIPL1	missense_variant	0	2.96	0.27	0.025	0.998	0.313	1.129	3.655	
17:7466469:A/G	NM_015670.5:c.76A>G(p.Ser26Gly)	no	.	2/92	2/46	0/102	0/51	D	P	P	SENP3,SEI	missense_variant	0	3.58	0.878	0.996	0.971	0.46	1.975	2.387	
17:8141511:C/T	NM_025099.5:c.485G>A(p.Arg162His)	no	rs1999660[illegible]	2/92	2/46	0/102	0/51	T	P	B	CTC1	missense_variant	0.000015	2.46	0.368	0.997	0.997	-2.069	0.161	0.497	
17:11684403:A/T	NM_001372.3:c.7630A>T(p.Ile2544Phe)	no	.	2/92	2/46	0/102	0/51	D	D	D	DNAH9	missense_variant	0	5.08	0.993	1	1	0.519	2.043	3.701	
17:16336966:G/A	NM_016113.4:c.2068G>A(p.Val690Ile)	no	rs54352943	2/92	2/46	0/102	0/51	T	P	B	TRPV2	missense_variant	0.000096	5.79	0.116	0.182	1	0.65	2.751	5.994	
17:17919916:G/C	NM_001130090.1:c.1503G>C(p.Glu501Asp)	no	.	2/92	2/46	0/102	0/51	D	P	B	LRRC48	missense_variant	0	2.36	0.856	1	1	0.655	0.783	1.143	
17:18923176:TT/-	NM_152351.4:c.1610_1611delTT(p.Phe537CysfsTer23)	no	.	2/92	2/46	0/102	0/51	.	.	.	SLC5A10	frameshift_variant	0	.	.	.	.	.	.	.	
17:26919577:G/A	NM_006461.3:c.685C>T(p.Arg229Cys)	no	rs5527373[illegible]	2/92	2/46	0/102	0/51	D	B	B	SPAG5	missense_variant	0.000086	-1.34	0.1	0	0.016	0.655	-0.204	-0.416	
17:28651866:G/A	NM_206832.1:c.608C>T(p.Thr203Met)	no	rs10557112	2/92	2/46	0/102	0/51	T	P	B	TMIGD1	missense_variant	0	-11.7	0.007	0	0	-1.99	-5.137	-7.22	
17:39118451:T/G	NM_213656.3:c.959A>C(p.Asn320Thr)	no	.	2/92	2/46	0/102	0/51	D	P	P	KRT39	missense_variant	0	1.76	0.538	0.073	0.998	0.533	0.427	3.101	
17:39253892:A/G	NM_031960.2:c.445T>C(p.Cys149Arg)	no	rs7626626[illegible]	2/92	2/46	0/102	0/51	D	P	P	KRTAP4-8	missense_variant	0.000091	3.3	0.92	0.109	1	0.369	1.276	3.132	
17:43112157:C/T	NM_024819.4:c.97G>A(p.Val33Met)	no	rs37126667	2/92	2/46	0/102	0/51	D	P	P	DCAKD	missense_variant	0.000099	1.14	0.541	0.73	0.993	-0.291	0.011	1.714	
17:49071175:T/C	NM_001130528.2:c.2348A>G(p.Asp783Gly)	no	.	2/92	2/46	0/102	0/51	D	D	D	SPAG9	missense_variant	0	5.93	0.95	1	1	0.533	2.263	7.622	
17:53026932:T/G	NM_005486.2:c.1236T>G(p.Ile412Met)	no	.	2/92	2/46	0/102	0/51	D	B	B	TOM1L1	missense_variant	0	-0.634	0.024	0	0	0.533	-0.208	-0.455	
17:54534700:G/C	NM_153228.2:c.1399G>C(p.Asp467His)	no	.	2/92	2/46	0/102	0/51	D	D	D	ANKFN1	missense_variant	0	6.17	0.998	1	1	0.655	2.941	9.873	
17:67211999:A/G	NM_080282.3:c.815T>C(p.Leu272Ser)	no	.	2/92	2/46	0/102	0/51	D	D	D	ABCA10	missense_variant	0	3.21	0.178	0.002	0.352	0.421	1.33	4.63	
17:72613429:C/A	NM_181449.2:c.216G>T(p.Arg72Ser)	no	.	2/92	2/46	0/102	0/51	D	P	P	CD300E	missense_variant	0	2.6	0.008	0.043	0	0.591	1.316	0.213	
17:80426764:A/G	NM_012336.3:c.377A>G(p.Lys126Arg)	no	rs74970797	2/92	2/46	0/102	0/51	D	B	B	NARF	missense_variant	0.00006	4.73	0.975	1	1	-0.347	1.027	6.866	
18:2890629:G/C	NM_032048.2:c.504G>C(p.Trp168Cys)	no	.	2/92	2/46	0/102	0/51	D	P	B	EMILIN2	missense_variant	0	-1.13	0.017	0	0.001	0.557	-0.096	0.993	
18:7033050:T/G	NM_005559.3:c.2096A>C(p.Asp699Ala)	no	.	2/92	2/46	0/102	0/51	D	B	B	LAMA1	missense_variant	0	3.67	0.101	0.393	0.999	-0.261	1.118	1.346	
18:8825194:G/A	NM_015210.3:c.3686G>A(p.Gly1229Asp)	no	rs77073954	2/92	2/46	0/102	0/51	D	B	B	SOGA2	missense_variant	0.000091	-1.22	0.527	0.002	0	-0.181	0.135	-0.663	
18:9258331:A/C	NM_015208.4:c.5066A>C(p.Gln1689Pro)	no	.	2/92	2/46	0/102	0/51	D	D	P	ANKRD12	missense_variant	0	5.33	0.887	0.994	0.998	0.533	2.141	3.543	
18:33702217:T/C	NM_012319.3:c.1157A>G(p.His386Arg)	no	.	2/92	2/46	0/102	0/51	T	P	B	SLC39A6	missense_variant	0	5.17	0.964	0.998	1	0.533	2.066	4.375	
18:42532741:C/T	NM_015559.2:c.3436C>T(p.Arg1146Trp)	no	rs7685366[illegible]	2/92	2/46	0/102	0/51	D	D	D	SETBP1	missense_variant	0.000086	5.15	0.992	0.997	1	0.561	1.505	3.651	
18:43678334:TCT/-	XM_005258282.1:c.28_30delAGA(p.Arg10del)	no	rs2019704[illegible]	2/92	2/46	0/102	0/51	.	.	.	ATP5A1	inframe_deletion	0	.	.	.	.	.	.	.	
18:47421483:T/A	NM_001080467.2:c.2873A>T(p.Glu958Val)	no	rs7460558[illegible]	2/92	2/46	0/102	0/51	D	D	D	MYO5B	missense_variant	0.00003	5.68	0.345	1	1	0.402	2.163	6.947	
18:56985594:G/A	NM_181654.3:c.101C>T(p.Pro34Leu)	no	.	2/92	2/46	0/102	0/51	D	P	P	CPLX4	missense_variant	0	5.36	0.741	0.977	1	0.655	2.666	9.107	
18:61233999:G/A	NM_080474.1:c.973G>A(p.Glu325Lys)	no	.	2/92	2/46	0/102	0/51	T	D	D	SERPINB1[illegible]	missense_variant	0	4.92	0.658	0.778	0	0.655	1.425	0.558	
18:72347020:G/T	NM_017757.2:c.4045G>T(p.Ala1349Ser)	no	rs7637824[illegible]	2/92	2/46	0/102	0/51	T	P	B	ZNF407	missense_variant	0.000015	1.67	0.891	0.806	0.999	0.655	2.754	1.807	
19:2097479:A/T	NM_001031735.2:c.355A>T(p.Asn119Tyr)	no	rs7561771[illegible]	2/92	2/46	0/102	0/51	D	D	P	IZUMO4	missense_variant	0.000061	4.22	0.928	1	1	0.459	1.868	4.621	
19:3751091:T/C	NM_004886.3:c.1661A>G(p.His554Arg)	no	rs1465840[illegible]	2/92	2/46	0/102	0/51	T	D	D	APBA3	missense_variant	0	4.85	0.087	0.988	1	0.459	1.808	5.731	
19:5654124:A/G	NM_001201338.1:c.1579A>G(p.Lys527Glu)	no	.	2/92	2/46	0/102	0/51	T	P	P	SAFB	missense_variant	0	5.34	0.487	0.565	1	0.46	2.145	6.074	
19:7685470:GCCC	NM_020196.2:c.2050_2057delGCCCGGGC(p.Ala684HisfsTer10)	no	.	2/92	2/46	0/102	0/51	.	.	.	XAB2	frameshift_variant	0	.	.	.	.	.	.	.	
19:8563391:C/A	NM_032152.4:c.1301G>T(p.Arg434Ile)	no	.	2/92	2/46	0/102	0/51	T	P	P	PRAM1	missense_variant	0	-5.81	0.699	0	0	-0.379	-1.187	-1.389	
19:13264095:G/A	NM_004907.2:c.95G>A(p.Arg32Gln)	no	.	2/92	2/46	0/102	0/51	D	D	D	IER2	missense_variant	0	4.88	0.988	0.999	1	0.462	2.253	6.579	
19:36235244:C/T	NM_001040425.1:c.211G>A(p.Val71Met)	no	rs7576259[illegible]	2/92	2/46	0/102	0/51	D	D	P	U2AF1L4	missense_variant	0.000015	1.91	0.251	0.989	0.986	-0.367	0.189	2.792	
19:41131903:C/T	NM_003573.2:c.4063C>T(p.Arg1355Cys)	no	rs7807798[illegible]	2/92	2/46	0/102	0/51	T	D	D	LTBP4	missense_variant	0.00009	4.47	0.533	0.766	0	0.557	2.301	0.829	
19:41268980:GTG/	NM_004596.4:c.600+3_600+6delGAGT(.)	no	rs76168157	2/92	2/46	0/102	0/51	.	.	.	SNRPA	splice_donor_variant	0.000049	.	.	.	.	.	.	.	
19:49964915:C/T	NM_153329.3:c.617C>T(p.Ala206Val)	no	rs7528690[illegible]	2/92	2/46	0/102	0/51	D	B	B	ALDH16A1	missense_variant	0.000096	4.52	0.201	0.011	0.002	0.585	2.464	1.044	
19:50037520:-/G	NM_020650.2:c.315dupG(p.Ile106AspfsTer45)	no	.	2/92	2/46	0/102	0/51	.	.	.	RCN3	frameshift_variant	0	.	.	.	.	.	.	.	
19:50826468:G/A	NM_004977.2:c.1742C>T(p.Pro581Leu)	no	rs7701144[illegible]	2/92	2/46	0/102	0/51	D	P	B	KCNC3	missense_variant	0	3.22	0.038	0.037	0.995	0.462	1.819	2.436	
19:51172180:G/A	NM_016148.2:c.3037C>T(p.Pro1013Ser)	no	rs5778043[illegible]	2/92	2/46	0/102	0/51	D	P	B	SHANK1	missense_variant	0	0.443	0.976	0.037	0.075	0.416	0.289	1.18	
19:52537143:A/C	NM_014650.2:c.1789T>G(p.Tyr597Asp)	no	.	2/92	2/46	0/102	0/51	D	D	P	ZNF432	missense_variant	0	1.92	0.992	0.959	0.001	0.533	0.354	1.398	
19:54696153:G/A	NM_024075.3:c.674G>A(p.Arg225Lys)	no	.	2/92	2/46	0/102	0/51	T	P	B	TSEN34	missense_variant	0	1.18	0.672	0.988	0.954	0.561	0.491	3.253	
19:58850415:G/T	NM_181846.2:c.1199G>T(p.Arg400Leu)	no	.	2/92	2/46	0/102	0/51	D	D	P	ZSCAN22	missense_variant	0	1.87	0.007	0.435	0.418	-0.253	0.464	3.421	
20:170218:G/C	NM_001037732.1:c.47C>G(p.Thr16Arg)	no	.	2/92	2/46	0/102	0/51	D	B	B	DEFB128	missense_variant	0	-0.0452	0.894	0.129	0.209	0.574	0.029	0.147	
20:2774867:C/T	NM_019609.4:c.2174G>A(p.Arg725His)	no	rs7677458[illegible]	2/92	2/46	0/102	0/51	D	D	P	CPXM1	missense_variant	0.000087	3.66	0.887	0.887	0.998	0.655	0.803	4.088	
20:3063736:G/A	NM_000490.4:c.209C>T(p.Ala70Val)	no	.	2/92	2/46	0/102	0/51	D	D	D	AVP	missense_variant	0	2.75	0.143	0.292	0.996	0.491	0.412	6.285	
20:3641753:G/A	NM_022139.3:c.230C>T(p.Pro77Leu)	no	.	2/92	2/46	0/102	0/51	D	D	D	GFRA4	missense_variant	0	4.31	0.973	0.996	1	0.555	2.233	5.031	
20:17622539:G/A	NM_001042576.1:c.788C>T(p.Ala263Val)	no	rs7601927[illegible]	2/92	2/46	0/102	0/51	T	P	B	RRBP1	missense_variant	0.000061	4.14	0.997	0.922	0.958	0.655	1.594	4.417	
20:23345861:G/T	NM_022482.3:c.841G>T(p.Gly281Cys)	no	.	2/92	2/46	0/102	0/51	D	P	B	GZF1	missense_variant	0	3.02	0.074	0.037	0.427	0.552	1.005	0.851	
20:44279139:C/T	NM_147197.2:c.100+1G>A(.)	no	.	2/92	2/46	0/102	0/51	.	.	.	WFDC11	splice_donor_variant	0	0.422	0.064	0.003	0.001	0.563	0.12	-0.05	
20:57429696:-/TGA	NM_001077490.1:c.1207_1208insCGACTCCGGGGCGGCCCGTGACGCCC	no	.	2/92	2/46	0/102	0/51	.	.	.	GNAS	inframe_insertion	0	.	.	.	.	.	.	.	
20:61470058:C/A	NM_001853.3:c.1809C>A(p.Asp603Glu)	no	.	2/92	2/46	0/102	0/51	T	D	D	COL9A3	missense_variant	0	4.36	0.367	1	1	0.555	2.484	0.812	
21:27938628:G/A	NM_052954.2:c.133C>T(p.Pro45Ser)	no	.	2/92	2/46	0/102	0/51	D	D	D	LOC10099[illegible]	missense_variant	0	5.05	0.981	0.957	1	0.591	2.731	4.405	
21:30442588:C/T	NM_006585.2:c.131G>A(p.Arg44His)	no	rs3695402[illegible]	2/92	2/46	0/102	0/51	D	D	D	CCT8	missense_variant	0	4.74	0.981	0.98	1	0.561	2.631	5.558	
21:34053882:C/A	NM_203446.2:c.1394G>T(p.Arg465Leu)	no	.	2/92	2/46	0/102	0/51	D	B	B	SYNJ1	missense_variant	0	5.83	0.998	0.997	1	0.585	2.756	7.778	
21:35772629:C/T	XM_005261086.1:c.742G>A(p.Gly248Arg)	no	.	2/92	2/46	0/102	0/51	D	.	.	LOC10192[illegible]	missense_variant	0	4.62	0.355	0.241	0.048	0.555	1.453	2.246	
21:44486423:A/C	NM_000071.2:c.381T>G(p.Ile127Met)	no	.	2/92	2/46	0/102	0/51	D	P	D	CBS	missense_variant	0	-10.5	0.706	0.006	0	-0.331	-4.369	-4.468	
22:19165667:G/A	NM_005984.3:c.181C>T(p.Pro61Ser)	no	.	2/92	2/46	0/102	0/51	T	P	B	SLC25A1	missense_variant	0	4.77	0.997	0.721	1	0.549	2.183	9.396	
22:19175545:TTG/-	NM_007098.3:c.4380_4382delCAA(p.Asn1460del)	no	rs78203782	2/92	2/46	0/102	0/51	.	.	.	CLTCL1	inframe_deletion	0.000061	.	.	.	.	.	.	.	
22:19961187:G/A	NM_001670.2:c.2218C>T(p.Arg740Trp)	no	.	2/92	2/46	0/102	0/51	D	D	P	ARVCF	missense_variant	0	2.34	0.831	0.981	0.983	0.491	0.592	1.918	
22:24124545:-/C	NM_005940.3:c.1210dupC(p.Arg404ProfsTer25)	no	.	2/92	2/46	0/102	0/51	.	.	.	MMP11	frameshift_variant	0	.	.	.	.	.	.	.	
22:24124548:G/A	NM_005940.3:c.1211G>A(p.Arg404Gln)	no	rs78072904	2/92	2/46	0/102	0/51	T	D	P	MMP11	missense_variant	0.000061	1.45	0.977	0.613	0.992	0.585	0.661	2.211	
22:24124556:G/C	NM_005940.3:c.1219G>C(p.Asp407His)	no	.	2/92	2/46	0/102	0/51	D	P	P	MMP11	missense_variant	0	1.6	0.967	0.67	0.997	0.585	0.752	3.177	
22:29886685:G/A	NM_021076.3:c.3056G>A(p.Gly1019Glu)	no	.	2/92	2/46	0/102	0/51	D	B	B	NEFH	missense_variant	0	1.22	0.192	0.991	0.222	-0.314	0.382	0.413	
X:12904286:CTA/-	NM_016562.3:c.661_663delACT(p.Thr221del)	no	rs7633313[illegible]	2/92	2/46	0/102	0/51	.	.	.	TLR7	inframe_deletion	0.000021	.	.	.	.	.	.	.	
X:15365439:A/G	NM_004469.4:c.785T>C(p.Met262Thr)	no	.	2/92	2/46	0/102	0/51	T	P	B	PIR-FIGF,F	missense_variant	0	3.92	0.914	0.994	1	0.486	1.822	2.722	
X:21675188:G/C	NM_153270.1:c.719C>G(p.Ser240Cys)	no	rs36989114	2/92	2/46	0/102	0/51	D	D	P	KLHL34	missense_variant	0	3.76	0.057	0.182	1	0.422	0.914	4.703	
X:101970104:A/T	NM_138437.5:c.307A>T(p.Arg103Trp)	no	rs7470137[illegible]	2/92	2/46	0/102	0/51	D	D	P	ARMCX5-G	missense_variant	0	1.81	0.326	0.76	0	0.486	0.617	-0.291	
X:149931118:G/-	NM_003828.2:c.1915delG(p.Val639TrpfsTer39)	no	.	2/92	2/46	0/102	0/51	.	.	.	MTMR1	frameshift_variant	0	1.94	0.466	0.999	1	0.529	0.408	2.079	
